# Integrative histopathological and immunophenotypical characterisation of the inflammatory microenvironment in spitzoid melanocytic neoplasms

**DOI:** 10.1111/his.14259

**Published:** 2020-11-19

**Authors:** Lisa M Hillen, Hendrik L D Vandyck, Daphne J G Leunissen, Bianca T A de Greef, Francesca M Bosisio, Axel zur Hausen, Joost van den Oord, Véronique Winnepenninckx

**Affiliations:** ^1^ Department of Pathology GROW School for Oncology and Developmental Biology Maastricht University Medical Center Maastricht the Netherlands; ^2^ Department of Clinical Epidemiology and Medical Technology Assessment Maastricht University Medical Center Maastricht the Netherlands; ^3^ Laboratory for Translational Cell and Tissue Research (TCTR) University of Leuven KUL Leuven Belgium; ^4^ Department of Pathology University Hospitals Leuven Belgium

**Keywords:** spitzoid melanocytic neoplasm, Spitz tumour, Spitz naevus (SN), atypical Spitz tumour (AST), malignant Spitz tumour (MST), T lymphocyte, inflammation

## Abstract

**Aims:**

The role of inflammation in conventional cutaneous melanoma has been extensively studied, whereas only little is known about the inflammatory microenvironment and immunogenic properties of spitzoid melanocytic neoplasms. The composition of infiltrating immune cells and the architectural distribution of the inflammation, in particular, are still obscure. This is the first study, to our knowledge, to systematically characterise the inflammatory patterns and the leucocyte subsets in spitzoid melanocytic lesions.

**Methods and results:**

We examined 79 spitzoid neoplasms including banal Spitz naevi (SN, *n* = 50), atypical Spitz tumours (AST, *n* = 17) and malignant Spitz tumours (MST, *n* = 12) using histopathological analysis and immunohistochemistry. Spitzoid melanocytic lesions showed a high frequency (67.1%, *n* = 53 of 79) of inflammation. Four inflammatory patterns were identified according to architectural composition, distribution and intensity of inflammation. The majority of the inflammatory infiltrate corresponded to CD3^+^/CD8^+^ T lymphocytes (56.1%), followed by CD3^+^/CD4^+^ T cells (35.7%) and CD68^+^ histiocytes (20.3%). CD3^+^/TIA‐1^+^ cytotoxic T lymphocytes constituted 3.7% of inflammatory cells. Rarely, CD3^+^/ granzyme B^+^ cytotoxic T lymphocytes (2.7%) and CD138^+^ plasma cells (0.5%) were detected in the infiltrating immune cells. There was no significant difference in the inflammatory cellular composition among the spitzoid melanocytic subgroups (SN versus AST versus MST).

**Conclusion:**

Our findings demonstrate that Spitz tumours are highly immunogenic lesions. Inflammation with the presence of lymphocytic aggregates predominated in SN, but was not distinctive for this melanocytic category. A strong and intense inflammation was suggestive of an underlying malignancy. The infiltrating cytotoxic T lymphocyte subsets in Spitz tumours deserve further investigation in larger study cohorts to elucidate prognostic and immuno‐oncological therapeutic relevance.

## Introduction

Spitzoid melanocytic neoplasms represent uncommon lesions accounting for only approximately 1–2% of resected melanocytic proliferations,^1^ with an estimated incidence from 1.4 to 1.6 per 100 000.^2^ They are characterised by epithelioid and/or spindle‐shaped melanocytes localised in a stromal background of variable amounts of lymphocytes, blood vessels and sclerosis. Spitzoid melanocytic neoplasms are named in honour of Sophie Spitz, an American pathologist, who initially proposed the term ‘melanoma of childhood’ for these lesions.^3^, ^4^ Although frequently occurring in children,^1^, ^3^, ^5^ lesions can also develop later in life and the patient’s age represents a risk factor for malignant progression.^6^ According to the recently launched 4th edition of the World Health Organisation (WHO) 2018 classification of skin tumours, spitzoid neoplasms range from banal Spitz naevi (SN) to atypical Spitz tumour (AST) to finally highly proliferative and pleomorphic malignant Spitz tumour (MST), i.e. spitzoid melanoma.^7^

A significant subset of spitzoid lesions is accompanied by an inflammatory infiltrate, which is mainly composed of lymphocytes.^1^, ^2^, ^5^ This inflammatory infiltrate can be attributed to the host response mechanism or immune surveillance, which is defined as the immunological response of the host against neoplastic cells.^8^, ^9^, ^10^ A host immune response is observed on a regular basis in melanocytic neoplasms with infiltration of the lesion by tumour‐infiltrating leucocytes (TILs), i.e. lymphocytes, that may result in elimination of part or all of the melanocytic lesion.^11^, ^12^, ^13^ In benign melanocytic lesions a strong inflammatory response with immunologically mediated rejection of the naevus cells and circumferential depigmentation of surrounding keratinocytes is known as the ‘halo phenomenon’, and eponymous for halo naevi (HN).^7^ The phenomenon of the immunogenic host response is also known as regression, and may be categorised into three temporal stages: early, intermediate and late.^14^ Early regression is characterised by the presence of TILs with an intimate association and contact with tumour cells, thereby disrupting them. Intermediate and late regression result in partial or complete loss of malignant melanocytes and are characterised by immature (intermediate) and mature (late) dermal fibrosis, often accompanied by dermal melanophages, and a flattened epidermis with loss of rete ridges.^14^ The intensity of TILs in conventional cutaneous malignant melanoma (CMM) is categorised by different grading systems, the most popular being from Clark *et al*.^15^ with (i) absent, (ii) brisk or (iii) non‐brisk inflammatory response, or the more recently proposed four‐tiered grading system (0–3) from the Melanoma Institute of Australia (MIA).^14^, ^16^ For benign melanocytic lesions, no such scoring system exists.

While the host response, including the immunogenic environment (particularly T lymphocytes and macrophages), has been extensively studied in conventional primary cutaneous melanoma,^10^, ^12^, ^15^, ^17^, ^18^, ^19^, ^20^, ^21^ the host response with the immunogenic environment in spitzoid melanocytic neoplasms remains largely elusive. In particular, the character of infiltrating T lymphocytes is unknown. Using molecular profiling mRNA gene expression analysis of a series of spitzoid lesions, we recently identified differential expression of inflammation‐associated gene transcripts, and gene set enrichment analysis (GSEA) revealed up‐regulation of immunomodulatory pathways in SN compared to common naevocellular naevi (NCN), thereby suggesting a unique role of inflammation in spitzoid neoplasms.^22^, ^23^ Furthermore, the distribution of the inflammation has been suggested but not yet confirmed as a helpful diagnostic feature.^24^, ^25^, ^26^

However, the detailed composition and intensity of the inflammatory infiltrate in spitzoid melanocytic neoplasms, especially with regard to the various T lymphocyte subsets, and the possible significance of the infiltrate in disease progression is still obscure. We hypothesised that the inflammatory infiltrate in spitzoid melanocytic neoplasms is likely to represent an essential component of the tumour microenvironment. Subtyping and characterisation of tumour‐infiltrating immune cells might aid clearer understanding of the role of inflammation in the pathogenesis of these rare lesions. Interestingly, it is well known that, similar to MST, AST frequently metastasise to locoregional lymph nodes but, in contrast to MST, rarely if ever spread to distant organs.^27^, ^28^ The mechanism behind this paradoxical behaviour remains entirely unknown. It is challenging to distinguish the ambiguous AST category in comparison to the group of highly aggressive MST solely on the basis of molecular genetic differences in the tumour genome.^23^, ^29^, ^30^ Thus, one may speculate that the environmental immunological neighbourhood hosting the spitzoid melanocytic neoplasms might ultimately define the lesion’s biological behaviour with either arrest of metastatic potential in the locoregional lymph node or dissemination to distant sites.

This study aims to characterise the inflammatory microenvironment in spitzoid melanocytic neoplasms (*n* = 79) with routine histopathology and immunohistochemistry (IHC). We also compared our findings to other distinct diagnostic melanocytic categories, including NCN (*n* = 20), HN (*n* = 25), blue naevi (BN, *n* = 22) and CMM (*n* = 20). Our findings demonstrate that spitzoid melanocytic lesions represent highly immunogenic neoplasms. We immunophenotyped the inflammatory infiltrate with special emphasis on cytotoxic T lymphocyte subsets and found that the inflammatory cellular composition among the different spitzoid tumour stages (SN versus AST versus MST) was homogeneous. We identified four different inflammatory patterns (IPs) in spitzoid melanocytic neoplasms with varying architectural composition and intensity of inflammation. Inflammation with the presence of lymphocytic aggregates (IP2) predominated in SN, but was not distinctive for this melanocytic category, whereas a strong and intense inflammation (IP3) in a spitzoid melanocytic neoplasm was suggestive for an underlying malignancy.

## Material and methods

### Patients and Tissue Specimens

**Figure 1:**
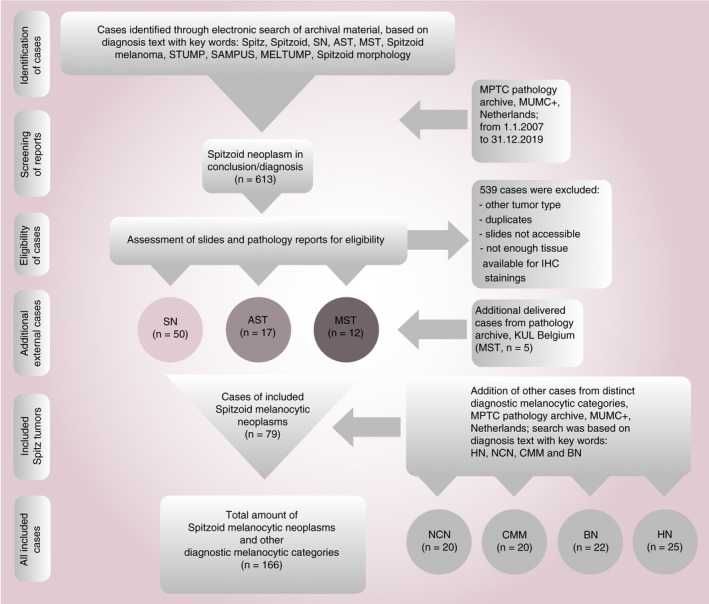
Course of selection of investigated spitzoid melanocytic neoplasms and additional diagnostic melanocytic categories. The flow chart demonstrates the course of selection for the investigated spitzoid melanocytic neoplasms, including Spitz naevi (SN), atypical Spitz tumour (AST) and malignant Spitz tumour (MST) as well as additional diagnostic melanocytic categories including naevocellular naevi (NCN), conventional cutaneous malignant melanoma (CMM), blue naevi (BN) and halo naevi (HN). AST, atypical Spitz tumour; FFPE, formalin‐fixed paraffin‐embedded; IHC, immunohistochemistry; KUL, Katholieke universiteit Leuven; MELTUMP, melanocytic tumours of uncertain malignant potential; MPTC, Maastricht pathology tissue collection; MST, malignant Spitz tumour; MUMC^+^, Maastricht University Medical Center; n, number; SAMPUS, superficial atypical melanocytic proliferation of unknown significance; SN, Spitz naevus; STUMP, spitzoid tumour of unknown malignant potential.

Formalin‐fixed paraffin‐embedded (FFPE) excision specimens of patients harbouring SN, AST or MST were retrieved from the archives of the Department of Pathology at the University of Leuven, KUL Belgium and the Maastricht Pathology Tissue Collection (MPTC) from the Department of Pathology, Maastricht University Medical Center (MUMC^+^), the Netherlands. The patients kindly provided written informed consent at hospital admission to the processing of tissue and personal data. All samples had been excised for diagnostic and therapeutic reasons. The patients were not reported to be immunocompromised at the time of tissue excision. All use of tissue and patient data was in agreement with the Dutch Code of Conduct for Observational Research with Personal Data and Tissue and in accordance with the Ethical Principles for Medical Research Involving Human Subjects (World Medical Association Declaration of Helsinki). Diagnoses were defined previously by histology in routine diagnostics and were confirmed by two experienced dermatopathologists (V.W., L.M.H.). The course of selection of cases is demonstrated in Figure 1. A total of 79 spitzoid melanocytic neoplasms were included in the analysis. Of these, 50 were SN, 17 were AST and 12 were diagnosed as MST (Table S1). A comprehensive overview of the clinical and histopathological criteria, which were applied to render the diagnosis of SN, AST or MST, are listed in Table S2.^7^, ^31^, ^32^, ^33^ In brief, SN were defined as benign melanocytic lesions composed of large epithelioid, oval or spindled melanocytes arranged in nests and/or fascicles without significant cytonuclear atypia.^33^ AST showed intermediate histological features of SN and MST with an increase in at least one worrisome histological feature, i.e. ulceration, size >5 mm, infiltrative growth into subcutaneous tissue with pushing margins, increased cytonuclear atypia, increase in cell density with confluent growth, more than two dermal mitoses, absence of junctional clefts, few or no Kamino bodies and more extensive pagetoid extension. MST was diagnosed on the basis of the following histological criteria: ulceration, asymmetrical architecture, infiltrative growth, severe and/or confluent cytonuclear atypia, dermal mitoses especially with deep dermal localisation, pushing borders, epidermal effacement and pagetoid extension. When rendering AST or MST as the diagnostic category, the case was additionally sent to an outside laboratory for external consultation. In order to compare the distribution of IPs, which were identified in the spitzoid melanocytic neoplasms, other distinct benign diagnostic melanocytic categories HN (*n* = 25), BN (*n* = 22), NCN (*n* = 20) and CMM (*n* = 20) were investigated. Clinical data, microscopic features, IP score, clinical and pathology follow‐up, state of local recurrence and other cutaneous pathology of these patients is provided in Table S1. For classification and staging, the 2016 TNM classification (8th edition) from the Union for International Cancer Control (UICC) was used in this study. The diagnosis of HN, BN, NCN and CMM was made according to the established criteria from the WHO 2018.^7^ A summary of the descriptive statistics for these cases is given in Table 1 and Table S4. The study was approved by the Maastricht Ethic Committee (MEC) of the University of Maastricht, the Netherlands and by the Institutional Review Board of the University Hospitals of Leuven, Belgium (project number S 59659).

**Table 1:** Summary of descriptive statistics for clinical data, histopathology characteristics and IPs including SN, AST, MST and other melanocytic diagnostic categories (HN, BN, CMM and NCN)

Melanocytic diagnostic category	SN	AST	MST	HN	BN	NCN	CMM
Study number (*n*)	50	17	12	25	22	20	20
Age (years)							
Mean	25.4	32.3	47.2	30.6	51.7	48.6	68.6
SEM	1.7	4.1	6.6	4.4	4.4	3.5	3.2
Median	24.0	26.0	49.5	28.0	49.5	52.5	73.0
SD	12.0	16.8	22.7	18.4	20.5	15.5	14.3
Minimum	1	13	10	10	19	21	32
25th centile	19.0	23.0	32.5	17.75	38.0	33.0	66.5
75th centile	31.3	37.0	59.0	36.0	68.75	58.25	79.0
Maximum	55	71	86	82	84	72	88
Gender							
Female	34	15	5	13	12	8	11
Male	16	2	7	5	10	12	9
NA	0	0	0	7	0	0	20
Anatomical site							
Upper extremities	11	5	4	0	8	1	4
Lower extremities	19	4	3	0	4	0	4
Corpus	9	7	3	16	3	18	7
Head/neck	10	1	2	2	7	1	5
NA	1	0	0	7	0	0	0
Histological features							
Junctional	7 (14%)	0	0	0 (0%)	0 (0%)	2 (10%)	0 (0%)
Dermal	5 (10%)	0	0	1 (4%)	22 (100%)	2 (10%)	0 (0%)
Compound	38 (76%)	17 (100%)	12 (100%)	25 (95%)	0 (0%)	16 (80%)	20 (100%)
Lesion diameter (mm)							
Mean	4.3	5.6	7.2	5.5	4.7	6.7	13.2
SEM	0.2	0.7	0.8	0.6	0.6	0.6	2.1
Median	4.0	5.0	6.0	5.0	4.0	6.0	9.5
SD	1.7	2.7	2.9	2.4	2.9	2.7	9.3
Minimum	1.8	2.5	5.0	2.0	1.0	2.0	3.0
25th centile	3.0	4.0	5.8	4.0	3.0	4.8	6.0
75th centile	5.0	7.0	7.3	7.0	5.5	8.3	18.5
Maximum	9.0	13.0	15.0	11.0	15.0	12.0	32.0
IP score							
IP0	4 (8%)	0 (0%)	1 (8.3%)	0 (0%)	18 (81.8%)	3 (15%)	1 (5%)
IP1	11 (22%)	5 (29.4%)	5 (41.7%)	0 (0%)	4 (18.2%)	10 (50%)	7 (35%)
IP2	33 (66%)	7 (41.2%)	3 (25%)	0 (0%)	0 (0%)	6 (30%)	7 (35%)
IP3	2 (4%)	5 (29.4%)	3 (25%)	25 (100%)	0 (0%)	1 (5%)	5 (25%)
Mean follow‐up (years)							
Mean	4.6	3.8	5.2	6.5	1.2	1.3	1.7
SEM	0.3	0.6	1.2	0.9	0.1	0.1	0.1
Median	4.0	3.0	4.0	7.0	1.0	1.0	2.0
SD	2.3	2.4	4.0	4.3	0.4	0.6	0.5
Minimum	1	1	1	1	1	1	1
25th centile	3.0	3.0	1.8	2.0	1.0	1.0	1.0
75th centile	6.0	5.0	8.3	10.0	1.0	1.3	2.0
Maximum	12	11	12	13	2	3	2
Local recurrence							
Yes	1 (2%)	1 (5.9%)	0 (0%)	0 (0%)	0 (0%)	0 (0%)	4 (20%)
No	49 (98%)	16 (94.1%)	12 (100%)	18 (72%)	22 (100%)	20 (100%)	16 (80%)
NA	0 (0%)	0 (0%)	0 (0%)	7 (28%)	0 (0%)	0 (0%)	0 (0%)

AST, Atypical Spitz tumour; BN, Blue naevus; HN, Halo naevus; CMM, Cutaneous malignant melanoma; mm, Millimetre; MST, Malignant Spitz tumour; *n* = Number; NA, Not available; NCN, Naevocellular naevus; SEM, Standard error of the mean; SD, Standard deviation; SN, Spitz naevus.

### Immunohistochemistry

Serial sections of the specimens were used for haematoxylin and eosin (H&E) staining and the most representative section for each case was selected for IHC investigation. IHC staining was conducted on formalin‐fixed paraffin‐embedded (FFPE) sections on a Dako Autostainer Link 48 using the EnVision FLEX Visualization Kit K8008 (Dako, Glostrup, Denmark), according to standard diagnostic routine protocols and the manufacturer’s instructions. Antibodies against CD3, CD4, CD8, CD68, CD138, TIA‐1 and granzyme B were used for the group of spitzoid melanocytic neoplasms in this study (for details see Table S3). On a limited number of cases (SN 39, SN 49, AST 52) we also screened for B lymphocytes using nuclear staining against PAX5 [Dako; ready‐to‐use (RTU) dilution] and membranous staining against CD20 (Dako; RTU dilution) and CD79a (Dako; RTU dilution). For the melanocytic diagnostic categories HN, BN, NCN and CMM, immunohistochemistry with antibody against CD3 was performed.

### Semiquantitative Analysis

The amount of TILs was quantified by three independent observers (V.W., H.L.D.V, L.M.H.) in at least four high‐power fields at ×40 magnification on H&E staining. Subsets of infiltrating leucocytes were quantified on the IHC stainings and given as the ratio of the total amount of inflammatory cells. Both the percentages of intra‐ and perilesional leucocytes were quantified, each in four randomly chosen regions that were representative for the whole lesion. Intralesional leucocytes were defined as leucocytes that were nested inside the tumour tissue in contact with tumour cells, while perilesional leucocytes were located outside the border of the tumour and not in contact with melanocytes. The percentage of TIA‐1^+^ and CD4^+^ lymphocytes with respect to the total number of inflammatory cells was quantified in eight high‐power fields with a magnification factor of ×40. As the number of CD138^+^ plasma cells and granzyme B^+^ lymphocytes was extremely low the whole lesion was screened, both intra‐ and perilesionally, at ×40 magnification. Only positive staining of cells with clear‐cut lymphocyte morphology was evaluated.

### Scoring System of the Inflammatory Patterns (IPs)

**Figure 2:**
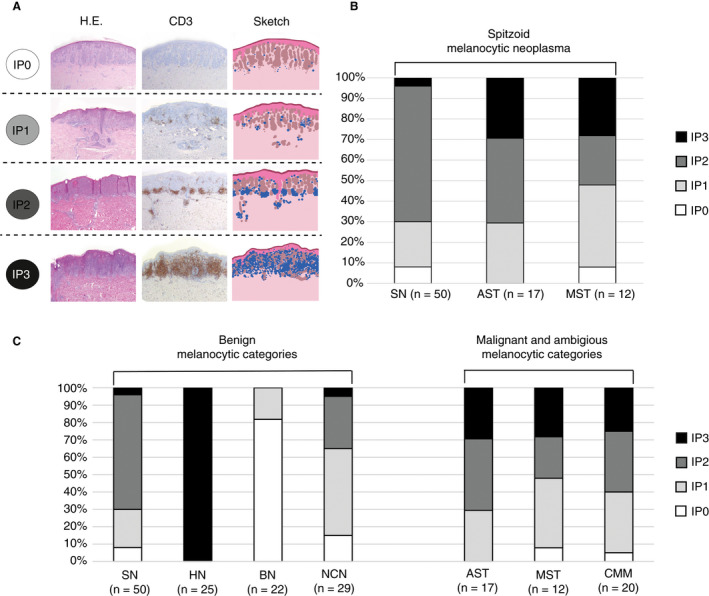
Inflammatory patterns (IPs) in spitzoid melanocytic neoplasms and comparison to different distinct melanocytic diagnostic categories. **A**, Segregation into four inflammatory patterns (IPs) in spitzoid melanocytic neoplasms with IP0 score (first row), IP1 score (second row), IP2 score (third row) and IP3 score (fourth row). Each row shows a spitzoid melanocytic lesion with haematoxylin &amp; eosin (H&amp;E) staining and a sketch (right images) highlighting the skin with epidermis and dermis (rose), inflammatory cells (blue) and the spitzoid melanocytic lesion (brown). The four IPs are classified according to: (IP0) without increase in number of detectable intra‐ or perilesional inflammatory cells on H&amp;E and in CD3 IHC staining; (IP1) with a sparse amount of intralesional CD3^+^ lymphocytes and scattering of the inflammatory cells in an isolated manner without formation of aggregates; (IP2) with sparse intralesional inflammation which is supplemented by secondary architectural structures consisting of nodular aggregates of perilesional CD3^+^ lymphocytes and localisation at the base or the lateral margin of the lesion; (IP3) is characterised by strong and diffuse intralesional as well as perilesional inflammation resembling the so‐called halo phenomenon. In IP3 there is diffuse formation of clusters and sheets of inflammatory cells. **B**, The clustered stacked column diagram shows the distribution of the four different IPs according to the investigated subcategories of spitzoid melanocytic neoplasms (*n* = 79). The detected IPs for the Spitz naevus cohort (SN, *n* = 50) are shown on the left, for atypical Spitz tumours (AST, *n* = 17) in the middle and for malignant Spitz tumour cases (MST, *n* = 12) on the right. **C**, The clustered stacked columns show the distribution of IPs for spitzoid melanocytic neoplasms (SN, AST and MST) in relation to IPs of other distinct benign melanocytic diagnostic categories (*n* = 87) including halo naevi (HN, *n* = 25), blue naevi (BN, *n* = 22), common naevocellular naevi (NCN, *n* = 20) and the malignant diagnostic category cutaneous malignant melanoma (CMM, *n* = 20).

To categorise the inflammation in the spitzoid melanocytic neoplasms in detail, a scoring system was established, thereby assessing the intensity of inflammation, the distribution of the inflammatory cells with respect to the spitzoid melanocytes and their architectural composition (Figure 2A). The IP was scored on the H&E section in conjunction with the CD3 IHC staining. Four categories were defined, ranging from IP0 to IP3. In the IP0 category there were virtually no detectable intra‐ or perilesional inflammatory cells on H&E and in CD3 staining. In the IP1 category there was a sparse amount of intralesional CD3^+^ lymphocytes with a scattering of single inflammatory cells without formation of aggregates. IP2 showed a sparse intralesional inflammation which was supplemented by secondary architectural structures consisting of nodular aggregates of perilesional CD3^+^ lymphocytes. The inflammatory aggregates were predominantly situated at the base or the lateral margin of the spitzoid melanocytic lesion. The IP3 score was characterised by strong and diffuse intra‐ and perilesional inflammation with formation of prominent clusters and sheets of inflammatory cells.

### Statistical Analysis

Statistical analysis was performed with SPSS IBM statistics software (version 26; SPSS Inc., Chicago, IL, USA) and r (version 3.6.1, statistics package). Normal distribution of data was analysed with the Shapiro–Wilk test. To detect significant differences between groups (*n* ≥ 3), the Kruskal–Wallis test was applied. For groups (*n* = 2), the Mann–Whitney *U*‐test was used. As a *post‐hoc* test Bonferroni correction was used, one‐way analysis of variance (anova) was used as a non‐parametric test to compare the means of numerical variables. Fisher’s exact and χ^2^ tests were used for categorical variables. Using the Pearson’s and Spearman’s tests, the correlation between the different data groups was examined. Results were considered to be statistically significant for *P* < 0.05 (see also Table S4). To assess the interobserver variability, Cohen’s kappa (κ) coefficient was calculated.

## Results

### Patient Cohort

For each patient, clinical features (including gender, age, anatomical site of the excised lesion, follow‐up) and histological findings (with junctional, compound or dermal architecture, symmetry of the lesion, dominating melanocytic cell type, presence of cytonuclear atypia, mitotic activity, epidermal changes, pigmentation and largest dimension in millimetres) were recorded (Table 1 and Table S1). The SN group consisted of 34 female and 16 male patients, ranging in age between 1 and 55 years [mean = 25.4 years, standard error of the mean (SEM) ± 1.7 years, median 24.0 years, Table 1]. The 25th and 75th centiles are given in Table 1 for each diagnostic category. The AST group consisted of 15 female patients and two male patients (range = 13–71 years, mean = 32.3 years, SEM ± 4.1 years, median = 26.0 years, Table 1). The MST group contained five female and seven male patients (range = 10–86 years, mean = 47.2 years, SEM ± 6.6 years, median = 49.5 years, Table 1). Patients from the SN group tended to be younger in relation to patients from the MST group. Patients with the diagnosis of AST were between the mean SN and MST age groups. However, the differences in age between the SN versus AST versus MST groups were not significant (Table S4). There was also no significant gender difference between SN versus AST versus MST. The lesions were localised on the extremities, head and neck area or on the corpus (20 upper extremity, 26 lower extremity, 19 corpus, 13 head and neck area, Table 1). For one patient with a SN (SN 41) the exact localisation was unknown (Table 1 and Table S1). None of the anatomical sites correlated with a specific subgroup of spitzoid melanocytic neoplasms.

The majority of spitzoid melanocytic lesions (84.8%, *n* = 67 of 79) presented with a compound histological architecture [SN 38 of 50 (76%), AST 17 of 17 (100%), MST 12 of 12 (100%), Table 1 and Table S1]. Pure junctional (seven of 50, 14.0%) or dermal (five of 50, 10%) localisation was only present in the SN group. Lesion diameter in MST (mean = 7.2 mm, SEM ± 0.8 mm) was significantly (*P* < 0.004) larger compared to SN (mean = 4.3 mm, SEM ± 0.2 mm, Table 1 and Table S4). There was also a trend of increase in lesion diameter from SN to AST (mean = 5.6 mm, SEM ± 0.7 mm) and from AST to MST, but this failed to reach a statistically significant difference (*P* < 0.063 for SN versus AST and *P* < 0.107 for AST versus MST, Table S4). Mean follow‐up time of patients was 4.6 years (SEM ± 0.3 years, median = 4.0 years) for the SN group, 3.8 years (SEM ± 0.6 years, median = 3.0 years) for the AST group and 5.2 years (SEM ± 1.2 years, median = 4.0 years) for the MST group, showing no significant difference between the three groups (Table S4). Local recurrence of the lesion occured in 2% (one of 50 patients) in the SN group, in 5.9% (one of 17 patients) in the AST group and was absent (0%, none of 12 patients) in the MST group.

### Frequency of Inflammation and Inflammatory Patterns (IPs) in Spitzoid Melanocytic Neoplasms

Inflammation was particularly present in all spitzoid melanocytic neoplasms, with an IP2 or IP3 in 67.1% (53 of 79) of cases. When including the IP1 category, an inflammatory background was found in 93.7% (74 of 79) of spitzoid lesions (Table 1 and Table S1, Figure 2A, second to fourth row).

In only 6.3% (five of 79) of spitzoid melanocytic neoplasms, an inflammatory infiltrate was absent corresponding to an IP0 score (Figure 2A, first row). Of the five IP0 samples, four cases belonged to the group of banal SN (8%, four of 50) and one case belonged to the MST group (8.3%, one of 12; Table 1 and Table S1, and Figure 2B, left and right columns). There was an even distribution of IP0 among gender and the different age groups of patients with a spitzoid melanocytic neoplasm. Absence of inflammation on H&E staining was also confirmed by negative IHC staining against CD3, CD4, CD8, CD68, CD138, TIA‐1 and granzyme B.

An IP1 score was observed in 26.6% (21 of 79) of patients with a spitzoid melanocytic neoplasm. This inflammatory pattern was present with a frequency of 22% (11 of 50) in SN, 29.4% (five of 17) in AST and 41.7% (five of 12) in MST (Figure 2A, second row). There was a higher percentage of female patients in cases with an IP1 score (female = 76.2%, 16 of 21; male = 23.8%, five of 21), whereas the age distribution was regular.

An IP2 score was observed in 54.4% (43 of 79) of spitzoid melanocytic neoplasms and was most frequent in the SN group (66%, 33 of 50 of SN samples; Table 1 and Supporting information, Table S1, Figure 2A, third row, Figure 2B, left column). The IP2 score was present in 41.2% (seven of 17) of AST (Table 1 and Supporting information, Table S1, and Figure 2B, centre column) and in 25% (three of 12) of MST (Table 1 and Table S1, and Figure 2B, right column). Lesions with IP2 score showed a sparse diffuse intralesional inflammation in addition to nodular aggregates of perilesional inflammatory cells (Figure 2A, third row) that were predominantly localised at the base, as well as the lateral margins of the lesions. Similarly to the IP1 group, there were 62.8% (27 of 43) female and 37.2% (16 of 43) male patients in the IP2 group. The 20–30‐year‐old group of patients was most frequently present (44.2%, 19 of 43) in the IP2 group; 16.3% (seven of 43) of patients in the IP2 group were younger than 20 years and 39.5% (17 of 43) were aged between 30 and 71 years.

Lesions with an IP3 score were characterised by strong and diffuse intralesional as well as perilesional inflammation, with prominent clusters and sheets of inflammatory cells (Figure 2A, fourth row). A strong diffuse inflammatory infiltrate with an IP3 score was detected in a minority of spitzoid melanocytic lesions 12.7% (10 of 79). All IP3 samples belonged to the AST group (29.4%, five of 17) and MST (25.0%, three of 12), with the exception of two cases, which belonged to the SN group (4%, two of 50; SN14 and SN43, Table 1 and Table S1, and Figure 2B, all columns). The patient with the SN14 lesion was also known to harbour a dysplastic naevus in the clinical history (Table S1). There were predominantly female patients in the IP3 group (80%, eight of 10) and only 20% (two of 10) male patients. The two male patients with IP3 belonged to the MST group. The age distribution was balanced.

There was no correlation of IP scores with the anatomical localisation. The interobserver variability of scoring the IPs was good (κ = 0.896 for the spitzoid group of neoplasms, *n* = 79 and κ = 0.959 for all investigated melanocytic lesions, *n* = 166).

### Distribution of Inflammatory Patterns (IPs) in Spitzoid Melanocytic Neoplasms in Relation to Other Distinct Melanocytic Diagnostic Categories

The distribution of IPs in spitzoid melanocytic neoplasms was also investigated in relation to other distinct melanocytic diagnostic categories (*n* = 87). The clustered stacked column diagram in Figure 2C shows the distribution of IPs for spitzoid melanocytic neoplasms (SN, AST and MST) in relation to IPs of other distinct benign melanocytic diagnostic categories (HN, *n* = 25; BN, *n* = 22; NCN, *n* = 20; and CMM, *n* = 20). Clinical, histological and follow‐up data are provided in Table S1 and results from descriptive statistical analysis are listed in Table 1 and Table S4.

The IP0 score corresponding to an absence of an inflammatory response was largely present in the benign categories. IP0 was most frequent in BN (81.8%, 18 of 22), followed by NCN (15%, three of 20), but was rarely present in SN (8%, four of 50, Table 1 and Table S1). IP0 was absent in AST. In the malignant categories the IP0 pattern was present in a small number of MST (8.3%, one of 12) and CMM (5%, one of 20).

The IP1 pattern was observed most frequently in NCN (50%, 10 of 20), followed by MST (41.7%, five of 12), CMM (35%, seven of 20), AST (29.4%, five of 17), SN (22%, 11 of 50) and BN (18.2%, four of 22) and was absent in HN (0%, none of 25).

The IP2 category with small aggregates of inflammatory cells was present in the majority of SN (66%, 33 of 50). The IP2 pattern was observed in a markedly smaller number in NCN (30.0%, six of 20) and completely absent in HN (0%, none of 25) and BN (0% none of 22). In atypical and malignant spitzoid lesions the frequency of the IP2 pattern decreased from 41.2% (seven of 17) in AST to 25% (three of 12) in MST. The IP2 pattern was detected in 35% (seven of 20) of CMM. All spitzoid melanocytic diagnostic categories remained clearly beneath the number of IP2 detected in the SN group.

IP3 was present in all HN (100%, 25 of 25), whereas only 4% of SN (two of 50) and 5% (one of 20) of NCN showed an IP3 score. In BN, inflammation with an IP3 score was completely absent (0%, none of 25). AST (29.4%, five of 17) and the malignant categories MST (25%, three of 12) and CMM (25%, five 20) regularly showed a strong inflammatory response with an IP3 score.

Overall, the atypical category AST, and the malignant groups of MST and CMM showed a mixture of the IPs (IP1, IP2 and IP3) and infrequently absence of inflammation (IP0).

### Distribution and Intensity of Infiltrating Inflammatory Cells

**Figure 3:**
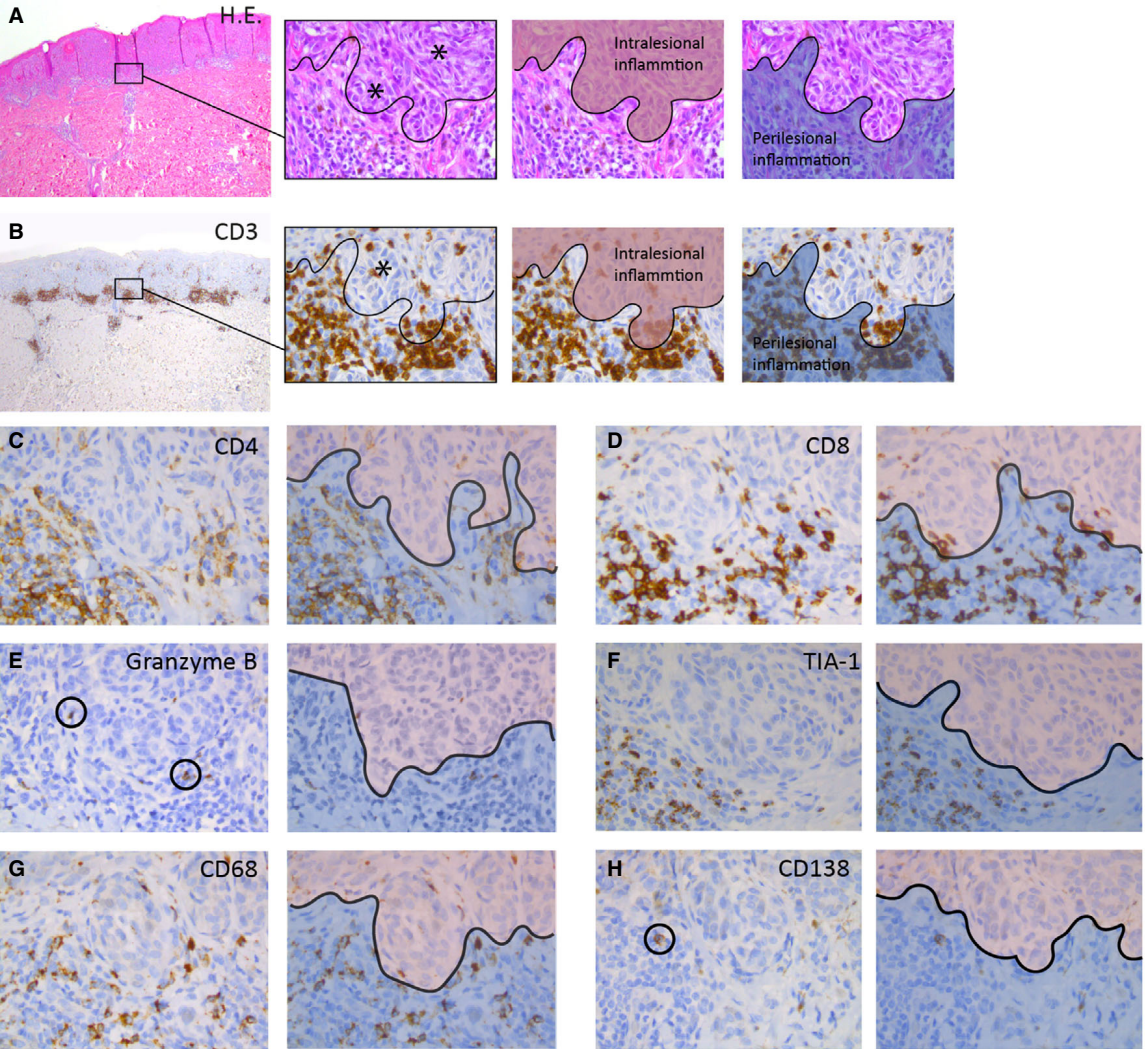
Immunohistochemical phenotypes in spitzoid melanocytic neoplasms. **A**, The left image shows a compound Spitz naevus (SN) used in this study with a symmetrical architecture and sharp lateral demarcation as well as circumscription towards the dermal depth. There is some intralesional inflammation as well as perilesional inflammatory aggregates at the base of the lesion. The second image from the left depicts a magnification from the left image and illustrates the two pathognomonic spitzoid cell types with epithelioid and spindle‐shaped melanocytes containing enlarged sharply demarcated nuclei with prominent nucleoli (black asterisks). The black curvilinear line demarcates the ‘grenz zone’ between intra‐ and perilesional inflammation. The two consecutive images accentuate the intralesional (highlighted in red) and perilesional (highlighted in blue) inflammatory areas. **B**, Left image with CD3 immunohistochemistry (IHC, brown staining) for the SN from (**A**). The three right images are magnifications from the left image and illustrate individual inflammatory CD3^+^ T cells in relation to the spitzoid epithelioid and spindle‐shaped melanocytes (blue‐stained nuclei). CD3^+^ T lymphocytes showed frequent peri‐ and intralesional presence (black asterisk, second image from the left). Intralesional inflammation is highlighted in red and perilesional areas are highlighted in blue in the two images from the right. The inflammatory pattern (IP) corresponds to an IP2 score (see also Figure 1A). **C**, The CD4 IHC staining depicts T helper lymphocytes and monocytes with a smudgy membranous accentuation (brown staining). CD4^+^ immune cells were frequently present in perilesional areas (highlighted with blue in the adjacent right image), but less often in intralesional zones (highlighted with red in the adjacent right image). **D**, There was a high amount of solitary infiltrating or clustering CD8^+^ T lymphocytes with circumscribed membranous staining (brown) with peri‐ (highlighted with blue in the adjacent right image) and intralesional localisation (highlighted with red in the adjacent right image). **E**, Rarely cytotoxic granzyme B^+^ T lymphocytes (encircled with black) were detected with granular cytoplasmic staining in perilesional (highlighted with blue in the adjacent right image) and intralesional (highlighted with red in the adjacent right image) localisation. **F**, TIA‐1 positive T lymphocytes with granular cytoplasmic staining (brown) were regularly present in the inflammatory infiltrate in perilesional areas (highlighted with blue in adjacent right image), but only rarely infiltrated within the individual spitzoid melanocytes (highlighted with red in adjacent right image). **G**, CD68^+^ histiocytes with smudgy cytoplasmic and membranous staining (brown) were presents in intralesional (highlighted with red in adjacent right image) and perilesional (highlighted with blue in adjacent right image) areas. **H**, Rarely a CD138^+^ plasma cell (encircled) with granular cytoplasmic staining and membranous accentuation (brown) was present in perilesional areas (highlighted with blue in adjacent right image) in the spitzoid melanocytic lesions. [Colour figure can be viewed at wileyonlinelibrary.com]

**Figure 4:**
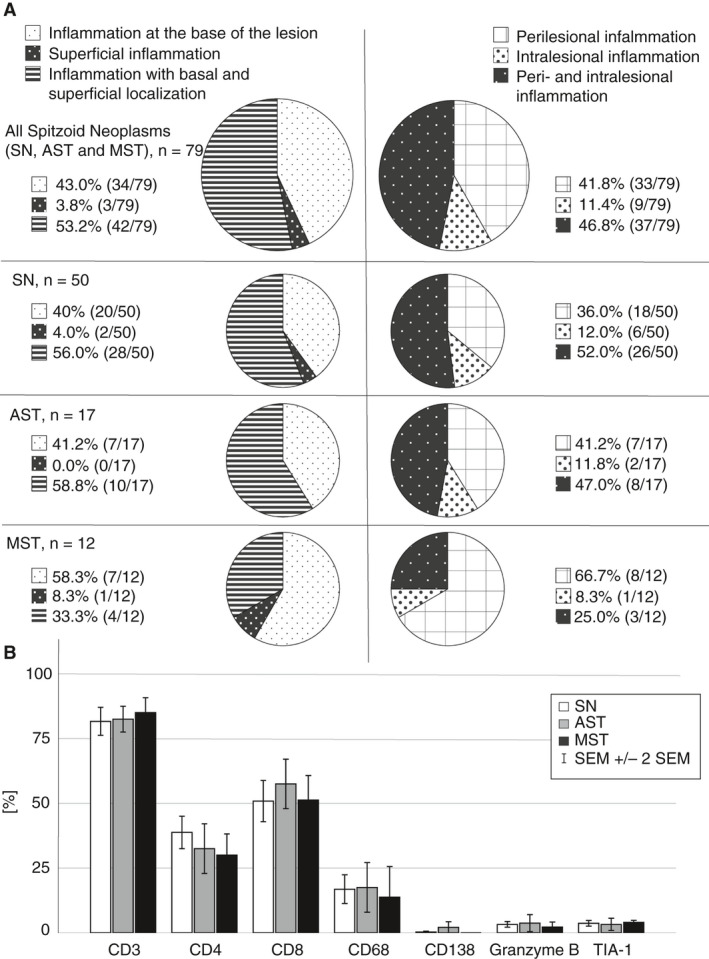
Distribution of inflammation in spitzoid melanocytic neoplasms and quantification of immunohistochemical phenotypes. **A**, The cake diagrams illustrate the architectural composition of the inflammatory infiltrate in spitzoid melanocytic neoplasms (*n* = 79, top row). The left column of cake diagrams differentiates proportion of neoplasms with superficial, basal or superficial and basal localisation of the inflammatory infiltrate. The right column of cake diagrams shows the results for segregation into intra‐, peri‐ or intralesional and perilesional localisation of inflammation. The cake diagrams in the second row correspond to the inflammatory distribution in the subgroup of Spitz naevi (SN, *n* = 50). The third row shows the results for atypical Spitz tumour (AST, *n* = 17) and the bottom row for malignant Spitz tumours (MST, *n* = 12). **B**, The amount of infiltrating inflammatory leucocytes with their immunohistochemical phenotypes is shown in white bars for Spitz naevi (SN), in grey bars for atypical Spitz tumours (AST) and in black bars for malignant Spitz tumours (MST). Error bars show ± 2 standard errors of the mean (SEM).

The majority of spitzoid melanocytic lesions (53.2%, 42 of 79) showed a diffuse distribution of the inflammatory infiltrate with superficial epidermal and basal dermal permeation (Figure 4A, top cake diagram on the left). Lymphocytes diffusely infiltrated between and around individual spitzoid melanocytes in the dermis, as well as melanocytic junctional nests (Figure 3A–F). In 43% (34 of 79) of investigated samples the inflammatory infiltrate was distributed in the dermal base of the lesion with sparing of superficial areas, whereas in 3.8% (three of 79) of spitzoid melanocytic neoplasms the inflammatory infiltrate was exceptionally present in superficial areas (Figure 4A, top cake diagram on the left). The inflammation showed intralesional and perilesional localisation in 48.8% (37 of 79) of spitzoid lesions (Figure 4A, top cake diagram on the right). In 41.8% (33 of 79) the inflammation was exceptionally present perilesionally and in 11.4% (nine of 79) the inflammation was detected only intralesionally. The distribution of infiltrating inflammatory cells for the subgroups SN, AST and MST is illustrated in Figure 4A (cake diagrams second, third and fourth rows). MST comprised the highest number of either a perilesional (66.7%, eight of 12) and/or an isolated basal (33.3%, four of 12) localisation of inflammation (Figure 4A, cake diagrams fourth row). Approximately half of SN and of AST showed a combination of inflammation with basal and superficial localisation (SN = 56%, 28 of 50, AST = 58.8%, 10 of 17) and/or with peri‐ and intralesional localisation (SN = 52%, 26 of 50, AST = 47%, eight of 17; Figure 4A, cake diagrams second and third rows).

### Immunohistochemical Phenotypes in the Inflammatory Infiltrate

The inflammatory cells were mainly composed of small lymphocytes admixed with histiocytic cells. The subsets of CD3^+^ T lymphocytes were studied with IHC antibodies against CD4, CD8, TIA‐1 and granzyme B (Figures 3A–H and 4B). CD3^+^ T lymphocytes (77.4%, Figure 4B) were found in aggregates, but also as individual infiltrating cells in close relation to the spitzoid epithelioid and spindle‐shaped melanocytes or in a perivascular localisation in the tumour stroma (Figure 3B). CD8^+^ T lymphocytes represented the major subset of infiltrating inflammatory leucocytes in spitzoid melanocytic neoplasms (56.1%, Figures 3D and 4B). CD4^+^ inflammatory T helper lymphocytes were also frequently present (35.7%, Figure 4B) within the inflammatory areas (Figure 3C). The mean ratio of CD4^+^/CD8^+^ lymphocytes was 0.72. In 3.7% of inflammatory cells there was granular cytoplasmic staining for TIA‐1 (Figures 3F and 4B). TIA‐1^+^ cytotoxic T lymphocytes were predominantly localised around spitzoid melanocytic cell nests, and only rarely infiltrated in between individual spitzoid melanocytes (Figure 3F). This contrasted with CD3^+^/CD8^+^ T lymphocytes which were found scattered within spitzoid melanocytic cell nests (Figure 3B,D). A fifth (20.3%) of inflammatory cells corresponded to CD68^+^ histiocytes in both peri‐ and intratumoural areas (Figure 4B). As shown in Figure 3G, CD68^+^ histiocytes were diffusely present in between the infiltrating T lymphocytes. A small subset of lymphocytes showed cytoplasmatic granular staining for the cytotoxic T cell marker granzyme B (2.7%, Figures 3E and 4B). Similarly, the number of CD138^+^ plasma cells was very low (0.5%) compared to the other leucocyte subsets (Figures 3H and 4B).

A limited number of cases were also investigated for the presence of B lymphocytes with antibodies against PAX5, CD20 and CD79a, but showed virtually no immunoreactive cells (data not shown).

There was no significant difference in the composition of infiltrating leucocyte subsets between the spitzoid melanocytic subgroups (SN versus AST versus MST, Figure 4B).

## Discussion

### Frequency of Inflammation and IPs in Spitzoid Melanocytic Neoplasms

Although inflammation is frequently observed in spitzoid melanocytic neoplasms,^1^, ^2^, ^34^, ^35^, ^36^ a more detailed characterisation and analysis of the distribution, composition and immunophenotypical characteristics of the inflammatory cells has only marginally been considered in previous series. Thus, the aim of this study was to more clearly characterise the inflammatory microenvironment in spitzoid melanocytic neoplasms. The results of the current study are the first, to date, assessing the inflammatory infiltrate in AST and MST in detail, and are the first to broadly characterise the inflammatory infiltrate in SN, AST and MST with immunohistochemical phenotyping. Our findings underline that spitzoid melanocytic lesions represent highly immunogenic neoplasms with 67.1% (53 of 79) of cases corresponding to an IP2 or IP3 score. Our data confirm observations from other groups that reported an inflammatory infiltrate to be present in 61%,^26^ 69%,^2^ 51%,^3^ 73%,^5^ 70%^1^ and 23%^35^ and in 75%^34^ of their studied cases (for overview see also Table 2). Berlingeri‐Ramos *et al*. observed in only a small proportion of SN (23%) an inflammatory infiltrate. The clear outlier nature of this study compared to other published series and our data has been conceded to reflect demographic differences in their studied Hispanic population.^35^ However, a statistical fluke is also possible, especially because that study does not provide a rationale for why Spitz lesions would have different immunogenicity in their cohort (i.e. Hispanic individuals) than other populations. Interestingly, they report that 9% of cases showed a lichenoid pattern of inflammatory cells. We observed isolated superficial inflammation in 3.8% (three of 79) of cases in our study. Of note, these previous series only considered SN in their investigations, but not AST and/ or MST.

**Table 2:** Chronological summary of studies in the literature assessing the inflammatory infiltrate in spitzoid melanocytic neoplasms

Frequency of infiltrate (%) and number of Specimens	IHC	Context of the study and comments	Type of spitzoid melanocytic lesion (SN, AST, MST)	Year	Reference
69% (146/211 cases)	NA	No reported meaningful pattern of distribution of inflammatory infiltrate	211 SN; 0 AST or MST	1977	^2^
100% (17/17 cases)^1^	CD8 TIA‐1 CD1a CD68 Ki‐67	Investigation of Spitz naevi with halo reaction. In combined tumours (9/17 cases) the symmetrical lymphocytic infiltrate was directed exclusively against the Spitz naevus component	17 SN^1^; 0 AST or MST	1997	^50^
73% (181/247 cases)	NA	Inflammation was conspicuous in 17 cases (6.9%) with lichenoid distribution, and disruption of melanocytic nests	247 SN; 0 AST or MST	2005	^5^
70% (243/349 cases)	NA	Grading of inflammatory intensity in light (161/349), moderate (64/349) and heavy (18/349) infiltration and assessment of distribution of inflammatory infiltrate with perivascular (207/349), diffuse (19/349) and band‐like (17/349) pattern	349 SN; 0 AST or MST	2009	^1^
23% (30/130 cases)	NA	Investigators concede that the low frequency of inflammation may reflect demographic differences in the studied population	130 SN; 0 AST or MST	2010	^35^
61% (42/69 cases)	NA^2^	Assessment of histomorphological diagnostic criteria of AST (*n* = 75) and their interobserver variability. For six cases data are unknown. Categorisation into patterns (1–3) indicating no, focal or dense lymphohistiocytic inflammation. Results of the categorisation are not presented in detail in the study. However, the presence of lymphoid aggregates was not distinctive for clinical behaviour of AST, as it was present in 61% (36/59) of non‐recurrent cases and in 60% (6/10) of recurrent cases	0 SN; 75 AST, 0 MST	2014	^2^
75% (24/32 cases)	NA	The inflammatory infiltrate was distributed in the central part of the base of the lesion in six lesions (19%), in the periphery of the lesion in five lesions (15.5%) and diffuse in 13 lesions (40.5%)	32 SN; 0 AST or MST	2015	^34^

AST, Atypical Spitz tumour; IHC, Immunohistochemistry; HN, Halo naevus; MST, Malignant Spitz tumour; NA, Not available; SN, Spitz naevus.

^1^ Harvell *et al*. investigated the subgroup of 17 HN of a cohort of 1286 spitzoid melanocytic neoplasms.^50^

^2^ Gerami *et al*. performed fluorescence *in‐situ* hybridisation (FISH) analysis targeting the original melanoma probe set Chr. 6p25, 6q23, 11q13 and Cep6 and the newer modified probe set targeting Chr. 6p25, 9p21, 11q13 and 8q24 on all 75 AST investigated in this study.^26^

The significantly (*P* < 0.004) smaller lesion diameter in SN (mean = 4.3 mm) compared to MST (mean = 7.2 mm) and the tendency to younger age of SN patients compared to MST patients is in line with data from the literature.^1^, ^2^, ^5^, ^6^

There are only few reports in the literature which have described the inflammatory composition in spitzoid melanocytic lesions. Requena *et al*. scored the intensity of the inflammation as light, moderate or heavy and the architectural distribution of the infiltrate with a perivascular, diffuse or band‐like superficial localisation.^1^ Others reported a lichenoid distribution with disruption of melanocytic nests in cases with conspicuous inflammation.^5^ According to Barnhill and colleagues, the distribution of the inflammatory infiltrate can be a helpful diagnostic feature with a tendency of perivascular inflammation or a diffuse symmetrical distribution in benign spitzoid lesions, in contrast to a more patchy pattern in atypical spitzoid lesions.^1^, ^25^

In our study, we observed differences in inflammatory composition, variation in intensity of inflammation, and heterogeneity in distribution of the inflammatory cells within the group of spitzoid melanocytic lesions. Thus, four different IPs were introduced, which represented an easily applicable synoptic scoring tool. Application of the IPs integrated parameters such as intensity of inflammation with architectural features including peri‐ and intralesional distribution as well as superficial and deep localisation of the inflammatory infiltrate. The IP0 score corresponded to the absence of detectable intra‐ or perilesional inflammatory cells on H&E and in CD3 IHC staining, whereas the IP2 and IP3 indicated the presence of inflammation. The CD3 IHC staining was extremely useful to detect the TILs, especially in the IP1 category, because it was sometimes difficult to appropriately segregate small round‐shaped type B naevus cells in the NCN group from lymphocytes purely on the H&E section. As stated above, in almost the whole group of spitzoid melanocytic lesions there was inflammation with an IP score >0. An IP0 score was only present in 6.3% (five of 79) of spitzoid melanocytic neoplasms. In contrast, the majority of BN were not inflamed with an IP0 of 81.8% (18 of 22).

The IP1 category was regularly seen in spitzoid melanocytic neoplasm (26.6%, 21 of 79). One may speculate that the IP1 category, defined as sparse infiltration of CD3^+^ lymphocytes but without formation of aggregates, might represent a transition category smouldering between the beginning or end‐phase of an inflammatory process. IP1 was also detected in CMM (35%, seven 20) with the highest number of IP1 scores in NCN (50%, 10 of 20). Only a small number of BN (18.2%, four of 22) showed a discrete lymphocytic inflammation with an IP1 pattern.

The IP2 score corresponded to sparse diffuse intralesional inflammation supplemented by secondary architectural structures, which consisted of nodular inflammatory aggregates. Among the three categories of spitzoid melanocytic neoplasms the IP2 pattern was most frequent in SN (66%, 33 of 50), with a predominance in female patients (62.8%, 27 of 43), and an accumulation in the 20–30‐year‐old patient group (44%, 19 of 43). However, although the IP2 pattern predominated in SN (66%, 33 of 50), with a decrease in AST (41.2%, seven of 17) and MST (25%, three of 12), this pattern was not specific with respect to the preeminence of the spitzoid melanocytic lesion. Furthermore, the IP2 score was not specific for spitzoid lesions, as it was also present in NCN (30%, six of 20) and CMM (35%, seven of 20). It might be that the IP2 category, with the formation of nodular lymphocytic aggregates, represents a more organised and temporally chronic phase of an inflammatory response.

In the IP3 category there was a strong and diffuse intra‐ and perilesional inflammation, with clustering and formation of inflammatory sheets. All investigated HN corresponded to an IP3 pattern (100%, 25 of 25), which is a characteristic hallmark of these melanocytic lesions.^7^ The IP3 pattern was significantly (*P* < 0.001) present more frequently in ambiguous and malignant lesions (AST, MST and CMM) compared to the benign melanocytic categories (SN, NCN and BN). The results were not significantly different (*P* < 0.161) for benign and malignant lesions when including the HN category. Thus, for diagnostic practice it can be deduced that an intense inflammation with an IP3 score represents a caveat, and is suggestive of an underlying atypia or malignancy. However, the IP3 score was not a discriminatory feature between the ambiguous AST category (29.4%, five of 17) and the malignant MST category (25%, three of 12) in this study. This is in line with findings from Gerami and colleagues, which also described that the presence of lymphoid aggregates was not specific to the clinical behaviour of AST, as it was present in 61% (36 of 59) of non‐recurrent cases and in 60% (six of 10) of recurrent cases in their study.^26^ A further exception of this observation represents the HN diagnostic melanocytic category. The phenomenon of more intense inflammation in higher‐grade spitzoid melanocytic lesions deserves further elucidation. Confirmation is necessary in a larger cohort in the future to evaluate its potential diagnostic relevance, as well as its possible prognostic and eventually immuno‐oncological therapeutic relevance.

### IP Score in Spitzoid Melanocytic Neoplasms in Relation to Established TIL Grading Systems

A systematic analysis with the categorisation of TILs in melanoma has been performed in numerous studies and the presence of TILs has been associated with a favourable prognosis,^17^, ^19^, ^37^, ^38^, ^39^, ^40^, ^41^ while other studies failed to demonstrate such an association.^42^, ^43^, ^44^ Therefore, the diagnostic significance of TILs in melanoma remains controversial (for review see Fu *et al*.^16^). For benign melanocytic lesions, a systematic analysis regarding the host response with the presence of TILs is not performed on a regular basis and, to our knowledge, no such classification systems exist, such as those that have been proposed for melanoma. However, one may ask how far the four identified IPs (IP0‐3) that assess the role of inflammatory cells in the tumour microenvironment of spitzoid melanocytic neoplasms can be related to well‐established TIL scoring systems. Thus, under conscious disregard that the majority of investigated melanocytic lesions from this study were benign, it is nonetheless of value to set the findings from this study in relation to well‐established TIL scoring systems for melanoma^14^, ^15^ and other cancer types (for review see Hendry *et al*.^45^).

The most popular classification system to assess TILs in melanoma was proposed more than 30 years ago by Clark *et al*.^15^ Three categories are used to divide the TIL infiltrate into ‘absent’, ‘non‐brisk’ and ‘brisk’ inflammation. ‘Absent’ is defined as the absence of lymphocytes or, if present, TILs are not directly apposed to melanoma cells. A ‘non‐brisk’ infiltrate is defined as a focal (either isolated, multifocal or segmental) TIL infiltrate. The ‘brisk’ category defines the TILs either involved in the entire base of the tumour or with diffuse infiltration of the entire malignancy.^10^, ^46^ The IP0 pattern from this study, with the absence of an inflammatory host response with respect to the melanocytic lesions, fits well with the traditional lymphocyte infiltration ‘absent’ pattern. The IP3 pattern, characterised by a strong and diffuse intra‐ and perilesional inflammatory host response, corresponds to the classical inflammatory pattern of a ‘brisk’ inflammatory host response. The ‘non‐brisk’ category from the grading system from Clark *et al*.^15^ was further subcategorised in the IP1 and IP2 group in our study. A more detailed separation of the ‘non‐brisk’ pattern into two groups was found to be useful to characterise spitzoid melanocytic lesions, as the IP2 category with the formation of nodular lymphocytic aggregates was particularly prominent in these lesions, especially in SN. The formation of ectopic lymph node‐like structures (TL‐ELN) or tertiary lymphoid structures (TLS) has also been described in other solid tumours (for review see Coppola and Mule^47^) and in melanoma.^20^, ^21^, ^48^, ^49^ It has been suggested that these structures correspond to an ongoing adaptive immune response within the melanoma microenvironment.^10^ However, one should be careful to deduce diagnostic decisions from the presence of these lymphoid nodular structures. These structures have been reported to be present in desmoplastic melanoma as well as desmoplastic sclerosing SN.^49^ Our data are in line with this observation, as we have detected an IP2 pattern in the whole spectrum of spitzoid melanocytic lesions (SN, AST and MST), as well as in conventional NCN and CMM, but not in the melanocytic diagnostic category of HN and BN.

As others have found, the TIL infiltrate may be difficult to grade using the ‘brisk’ and ‘non‐brisk’ categories in some cases, and due to the presence of subgroups of patients with a dense ‘non‐brisk’ TIL infiltrate with an excellent prognosis there has been an alternative, more recently proposed, grading scheme by the MIA for quantifying the presence of TILs.^14^ The grading system proposed by the MIA consists of grades 0–3 and is based on the density and the distribution of TILs in the dermis.^14^, ^46^ The density and the distribution are scored separately with three gradations (focal, multifocal or diffuse) and afterwards combined with one of the four degrees (0–3) using a cross table. Grade 0 describes an absence of TILs, grade 1 consists of a mild or moderate focal or mild multifocal infiltrate; grade 2 describes a marked focal, either a moderate or marked multifocal or a mild diffuse TIL infiltrate; and grade 3 depicts a moderate or marked diffuse TIL infiltrate.^14^ Thus, grade 0 corresponds to ‘absent’ inflammation and/or the IP0 score. Grades 1 and 2, if the presence of TILs is focal marked or multifocal moderate or marked, correspond to ‘non‐brisk’ inflammation. Grades 1–3 can correspond to ‘brisk’ inflammation, depending on the distribution and density of TILs, taking into account that the entire base of the tumour shows involvement. Grades 2–3 can also correspond to ‘brisk’ inflammation, depending on the density of TIL infiltrate and on condition that the vertical growth phase of the melanoma is diffusely permeated by TILs. Again, a comparison of the TIL scoring system from the MIA with the IP grading system from our study is difficult, as it corresponds mainly to melanomas with extended vertical growth. In our study the majority of lesion was benign, and in cases of malignancy there were 75% (nine of 12) of MST with T1 or T2 stage and only 25% (three of 12) cases with a T‐stage >2. Furthermore, nodular lymphocytic aggregates were not considered separately in the classification system from the MIA. Disregarding these aspects, the IP0 score corresponds to MIA grade 0 and the IP1 corresponds best to grade 1 if TILs are mildly and/or focally present. MIA grades 1, 2 and 3 might account for the IP2 pattern. The IP3 score is covered best by MIA grades 2 and 3.

### Immunohistochemical Composition of the Inflammatory Infiltrate

In the present study an IHC panel with antibodies against CD3, CD4, CD8, granzyme B, TIA‐1, CD68 and CD138 was used. With this marker combination we characterised the composition of the infiltrating immune cells with an emphasis on cytotoxic T lymphocyte subsets whose role is almost unexplored in spitzoid melanocytic lesions. In fact, only one study has immunophenotyped the inflammatory cells in Spitz tumours, more than 20 years ago.^49^ In this study Harvell *et al*. investigated a group consisting of 17 SN harbouring a halo reaction with IHC against CD8, TIA‐1, CD1a, CD68 and Ki‐67. Interestingly, in combined tumours consisting of a SN with either a banal common naevus or a congenital naevus (nine of 17 cases), the lymphocytic infiltrate was directed exclusively against the SN component. This is in line with our observations, where we detected the lymphocytic cells to be localised in close relation to individual spitzoid melanocytes or around nests of spitzoid cells. Of note, our series of cases did not contain combined or collision tumours.

In this study, the inflammatory cells were predominantly composed of CD3^+^/CD8^+^ T lymphocytes (56.1%), followed by CD4^+^ immune cells (35.7%) and CD68^+^ histiocytes (20.3%). This is in line with the observations of Requena *et al*.,^1^ who reported that the infiltrate in SN was mainly composed of lymphocytes with a smaller proportion of histiocytes. However, they did not consider the parameter ‘composition of the infiltrate’ specifically, so that their observations are based on pure morphological grounds without immunophenotyping of the infiltrate.

TIA‐1 expression was observed in 3.7% of the infiltrating immune cells in the spitzoid melanocytic neoplasms. TIA‐1 is a 17‐kDa cytoplasmic granule‐associated protein also designated as granule membrane protein of 17 kDa (GMP‐17),^51^ and is expressed in cells possessing cytolytic potential.^52^, ^53^ Harvell *et al*. reported higher percentages, with 40–50% of the lymphocyte population that expressed TIA‐1. These data may be based on their selection of cases with the subgroup of Spitz tumours harbouring a halo reaction,^50^ as halo naevi are well known to harbour predominantly TIA‐1^+^ cytotoxic T cells.^54^ The 2G9 monoclonal antibody, which was used in this study, has been reported to react with approximately 50–60% of CD8^+^ and fewer than 10% of CD4^+^ normal peripheral blood lymphocytes.^55^ TIA‐1 antibody has been reported to react with almost all monocytes and granulocytes.^51^ In our study, the TIA‐1^+^ cytotoxic T lymphocytes were predominantly present in the inflammatory infiltrate around the spitzoid melanocytic cell nests. The TIA‐1 protein is involved in Fas (CD95)‐mediated apoptosis.^51^, ^53^, ^56^ Harvell *et al*. reported that, in many cases, melanocytes with pyknotic and karyorrhectic nuclei were surrounded by TIA‐1‐positive lymphocytes.^50^ In our study, an evident increase of apoptotic melanocytic cells around the TIA‐1^+^ lymphocytes was not obvious on histomorphological grounds. However, it might be worthwhile to screen the lesions with apoptosis‐related markers in future studies.

The intracellular serine protease granzyme B is also involved in apoptosis, and initiates DNA fragmentation in the target cell.^57^, ^58^, ^59^ Both activated cytotoxic T lymphocytes and natural killer (NK) cells express granzyme B.^60^, ^61^, ^62^ We observed cytoplasmic granular expression for granzyme B (clone GrB‐7) in up to 2.7% of all infiltrating lymphocytes in our cases. In conventional melanomas, granzyme B‐positive cytotoxic T lymphocytes constitute 1–10% of the inflammatory infiltrate and are believed to be involved in tumour cell killing through apoptosis induction, which may ultimately result in melanoma regression.^63^, ^64^, ^65^ To our knowledge, there are no data in the literature on expression of granzyme B in the microenvironment of spitzoid melanocytic lesions.

In our study, CD138^+^ plasma cells were absent or rare (0.5%) in spitzoid lesions. This is in line with observations from Requena *et al*.,^1^ who did not find plasma cells in their cases on histomorphological grounds. Weedon *et al*.^2^ found plasma cells only in a small subset of spitzoid melanocytic neoplasms (six of 211), in whom five of these six cases were ulcerated. The relationship between ulceration and plasma cell infiltration has also been reported for conventional cutaneous melanoma.^66^ However, our study cohort did not harbour ulcerated lesions.

Finally, there was no significant difference in the immunophenotypical composition of infiltrating leucocyte subsets between the different tumour stages of spitzoid melanocytic neoplasms (SN versus AST versus MST). This is in line with observations from Harvell *et al*., who also found a homogeneous distribution among all lesions in their study.^50^

### Limitations of the Study

A limitation of the present study is that the data are insufficient to predict a possible prognostic implication, as they are not segregable by different end‐points with respect to local recurrence or N‐ or M‐stage. The mean follow‐up time for the spitzoid lesions was 4.6 years for SN, 3.8 years for AST and 5.2 years for MST. The majority of cases were benign (SN, *n* = 50 of 79). Local recurrence was only seen in two cases (SN38 and AST66). There was no manifestation of nodal disease in the examined AST (N stage = 0 in 17 of 17 lesions). None of the MST cases showed local recurrence, nodal disease or distant metastasis (N0M0 stage in 12 of 12 cases).

Another limitation is that the sample size of AST (*n* = 17) and MST (*n* = 12) is below the sample size for SN (*n* = 50). As spitzoid melanocytic neoplasms are uncommon lesions the ambiguous AST and the malignant MST category are extremely rare.^1^, ^2^ Thus, when considering small sample size this study actually represents a large number of AST and MST cases.

## Outlook and future research

We intend to perform multiplex IHC analysis with a large panel of antibodies (*n* ~ 30) on a larger cohort of spitzoid melanocytic lesions. This technique enables multiple sequential IHC analyses on the same section.^67^, ^68^ We thereby aim to more accurately characterise the identified cytotoxic T lymphocyte subsets. This may yield a clearer understanding of the inflammatory reaction evoked by Spitz tumours.

As stated earlier, decisions regarding the benign or malignant behaviour of a spitzoid melanocytic neoplasm are at first made by weighing the histopathological architecture and cytological features of the underlying lesion. Nonetheless, the inflammatory environment also provides valuable information. Although our study is limited by the relatively small number of cases for AST and MST our findings raise many further research questions. Might the cytotoxic population of T lymphocytes be an indicator for host response against the spitzoid melanocytic lesion? Is the T lymphocytic immune response a good prognostic feature such as in conventional melanoma? Is there a relationship between the inflammatory microenvironment and metastatic potential in high‐grade lesions? Further study of patients with these rare melanocytic lesions is required in a larger group with long‐term follow‐up data to better understand these evolving issues.

Finally, due to the rarity of spitzoid melanocytic neoplasms, this research field certainly represents a niche category in neoplastic dermatopathology. However, the relevance of a research question and research work should not be based purely upon the quantitative number of patients affected by a disease. The research should, rather, be evaluated on the potential impact on understanding of the pathophysiology of the disease, thereby deepening our knowledge of disease in general.

## Conclusions

This study provides new insights into the inflammatory microenvironment of spitzoid melanocytic neoplasms and characterises the infiltrating leucocyte immunophenotypes, with emphasis on cytotoxic T cell subsets. The different detected IPs in spitzoid melanocytic neoplasms vary in architectural composition and intensity of inflammation. We conclude that inflammation with formation of lymphocytic aggregates (IP2) predominates in SN, but is not distinctive for this lesion category. Strong and intense inflammation (IP3) was significantly more frequently present in atypical and malignant lesions. Thus, a diffuse and strong inflammation in a spitzoid melanocytic neoplasm is suggestive of underlying malignancy. The highly immunogenic properties of these lesions may be of prognostic and therapeutic relevance, and should be elucidated in larger study cohorts in the future with long‐term follow‐up data, thereby eventually optimising immuno‐oncological treatment decisions and improving patient management.

## Author contributions

VW, JVO, AZH and LH designed the research question and established the study. AZH, VW, HV and LH provided patient tissue. LH, VW and HV performed the histopathology and immunohistochemistry expression analysis. VW, JVO, FB and LH designed and developed the outline and concept of the manuscript. BG, DL and LH performed the statistical analysis. LH edited the figures and tables. All authors wrote, discussed and commented the manuscript. All authors approved the final manuscript.

## Conflicts of interest

No potential conflicts of interest were disclosed by the authors.

## Supporting information


**Table S1.** Patient cohort with histopathology diagnosis, clinical data, microscopic features, score of inflammatory pattern (IP), clinical and pathology follow up, state of local recurrence and other cutaneous pathology in Spitz nevus (SN), atypical Spitz tumor (AST), malignant Spitz tumor (MST) as well as other melanocytic diagnostic categories comprising Halo nevus (HN), Blue nevus (BN), common nevocellular nevus (NCN) and conventional cutaneous malignant melanoma (CMM).
**Table S2.** Clinical and histopathological features of Spitzoid neoplasms.
**Table S3.** Overview of antibodies used for immunohistochemistry (IHC) staining.
**Table S4.** Statistical analysis of level of significance for the different diagnostic groups.

## Data Availability

The data that supports the findings of this study are available in the supplementary material of this article.
