# Supplementary material for: Integrative histopathological and immunophenotypical characterisation of the inflammatory microenvironment in spitzoid melanocytic neoplasms
Source: Histopathology. 2020 Nov 19;78(4):607–26. doi: 10.1111/his.14259 (PMC7894529; doi:10.1111/his.14259)
Supplement: Supplementary file 1 — Table S1. Patient cohort with histopathology diagnosis, clinical data, microscopic features, score of inflammatory pattern (IP), clinical and pathology follow up, state of local recurrence and other cutaneous pathology in Spitz nevus (SN), atypical Spitz tumor (AST), malignant Spitz tumor (MST) as well as other melanocytic diagnostic categories comprising Halo nevus (HN), Blue nevus (BN), common nevocellular nevus (NCN) and conventional cutaneous malignant melanoma (CMM). Table S2. Clinical and histopathological features of Spitzoid neoplasms. Table S3. Overview of antibodies used for immunohistochemistry (IHC) staining. Table S4. Statistical analysis of level of significance for the different diagnostic groups. [file HIS-78-607-s001.docx]

**Supplemental Material**

**Table S1:** **Patient cohort with histopathology diagnosis, clinical data, microscopic features, score of inflammatory pattern (IP), clinical and pathology follow up, state of local recurrence and other cutaneous pathology in Spitz nevus (SN), atypical Spitz tumor (AST), malignant Spitz tumor (MST) as well as other melanocytic diagnostic categories comprising Halo nevus (HN), Blue nevus (BN), common nevocellular nevus (NCN) and conventional cutaneous malignant melanoma (CMM)**

| **Number of patient** | **Histopathology diagnosis** | **Age (y)** | **Gender** | **Anatomical site** | **Histological features** | **Maximal lesion diameter (mm)** | **Breslow invasion depth (mm)** | **IP Score** | **Follow up (y)** | **Local recurrence** | **Other cutaneous pathology** | **TNM** |
| --- | --- | --- | --- | --- | --- | --- | --- | --- | --- | --- | --- | --- |
| 1 | SN | 6 | F | Head/ Neck | Compound | 9 | - | 2 | 3 | No |  |  |
| 2 | SN | 24 | F | Lower Extremities | Compound | 3 | - | 2 | 12 | No |  |  |
| 3 | SN | 25 | F | Upper Extremities | Compound | 4 | - | 2 | 9 | No |  |  |
| 4 | SN | 24 | F | Lower Extremities | Compound | 5 | - | 2 | 8 | No |  |  |
| 5 | SN | 34 | F | Corpus | Junctional | 3 | - | 1 | 9 | No |  |  |
| 6 | SN | 14 | M | Lower Extremities | Compound | 7 | - | 2 | 8 | No |  |  |
| 7 | SN | 34 | M | Head/ Neck | Compound | 5 | - | 2 | 8 | No |  |  |
| 8 | SN | 26 | F | Lower Extremities | Compound | 4 | - | 1 | 8 | No |  |  |
| 9 | SN | 29 | F | Lower Extremities | Compound | 3 | - | 2 | 7 | No |  |  |
| 10 | SN | 45 | M | Head/ Neck | Compound | 4 | - | 2 | 7 | No |  |  |
| 11 | SN | 28 | F | Lower Extremities | Junctional | 6 | - | 1 | 7 | No |  |  |
| 12 | SN | 28 | F | Lower Extremities | Compound | 7 | - | 2 | 7 | No |  |  |
| 13 | SN | 21 | F | Corpus | Junctional | 4 | - | 2 | 6 | No | Multiple NCN |  |
| 14 | SN | 28 | F | Upper Extremities | Compound | 3 | - | 3 | 6 | No | Dysplastic nevus |  |
| 15 | SN | 39 | F | Corpus | Compound | 4 | - | 2 | 6 | No |  |  |
| 16 | SN | 25 | F | Corpus | Dermal | 3 | - | 2 | 5 | No | BN |  |
| 17 | SN | 29 | F | Upper Extremities | Junctional | 2 | - | 2 | 2 | No |  |  |
| 18 | SN | 24 | M | Upper Extremities | Compound | 3 | - | 2 | 2 | No |  |  |
| 19 | SN | 33 | F | Corpus | Compound | 6 | - | 2 | 1 | No |  |  |
| 20 | SN | 32 | M | Upper Extremities | Compound | 7 | - | 2 | 1 | No |  |  |
| 21 | SN | 13 | F | Upper Extremities | Compound | 4 | - | 2 | 5 | No |  |  |
| 22 | SN | 21 | F | Upper Extremities | Compound | 3.5 | - | 1 | 5 | No |  |  |
| 23 | SN | 24 | F | Lower Extremities | Compound | 3.5 | - | 0 | 5 | No |  |  |
| 24 | SN | 18 | M | Head/ Neck | Compound | 4 | - | 2 | 5 | No |  |  |
| 25 | SN | 49 | F | Lower Extremities | Compound | 3.5 | - | 2 | 5 | No |  |  |
| 26 | SN | 22 | M | Lower Extremities | Junctional | 3.5 | - | 2 | 4 | No |  |  |
| 27 | SN | 19 | F | Upper Extremities | Compound | 6 | - | 1 | 4 | No |  |  |
| 28 | SN | 9 | M | Lower Extremities | Compound | 6 | - | 0 | 4 | No |  |  |
| 29 | SN | 34 | F | Lower Extremities | Dermal | 3 | - | 1 | 4 | No |  |  |
| 30 | SN | 18 | M | Head/ Neck | Compound | 1.8 | - | 1 | 4 | No |  |  |
| 31 | SN | 19 | F | Lower Extremities | Compound | 2.5 | - | 1 | 4 | No |  |  |
| 32 | SN | 28 | F | Head/ Neck | Compound | 3.2 | - | 2 | 4 | No |  |  |
| 33 | SN | 21 | F | Upper Extremities | Dermal | 4 | - | 2 | 3 | No |  |  |
| 34 | SN | 20 | F | Head/ Neck | Compound | 3.6 | - | 1 | 3 | No |  |  |
| 35 | SN | 55 | F | Lower Extremities | Dermal | 4 | - | 2 | 3 | No |  |  |
| 36 | SN | 13 | F | Corpus | Compound | 8 | - | 0 | 3 | No |  |  |
| 37 | SN | 22 | M | Lower Extremities | Compound | 3.5 | - | 2 | 3 | No |  |  |
| 38 | SN | 48 | M | Corpus | Compound | 2.5 | - | 2 | 3 | Yes |  |  |
| 39 | SN | 12 | M | Head/ Neck | Compound | 2 | - | 2 | 3 | No |  |  |
| 40 | SN | 1 | F | Head/ Neck | Junctional | 3 | - | 1 | 3 | No |  |  |
| 41 | SN | 6 | F | NA | Compound | 3 | - | 2 | 3 | No |  |  |
| 42 | SN | 45 | M | Corpus | Compound | 4 | - | 2 | 3 | No |  |  |
| 43 | SN | 6 | F | Head/ Neck | Dermal | 6 | - | 3 | 3 | No |  |  |
| 44 | SN | 28 | M | Upper Extremities | Compound | 5 | - | 2 | 3 | No |  |  |
| 45 | SN | 50 | F | Lower Extremities | Compound | 7 | - | 2 | 3 | No |  |  |
| 46 | SN | 24 | M | Lower Extremities | Compound | 5 | - | 2 | 3 | No |  |  |
| 47 | SN | 13 | F | Corpus | Compound | 7 | - | 1 | 3 | No |  |  |
| 48 | SN | 25 | F | Upper Extremities | Compound | 5 | - | 0 | 3 | No |  |  |
| 49 | SN | 37 | M | Lower Extremities | Junctional | 2.2 | - | 2 | 2 | No |  |  |
| 50 | SN | 22 | M | Lower Extremities | Compound | 3.5 | - | 2 | 4 | No |  |  |
| 51 | AST | 22 | F | Corpus | Compound | 7 | - | 3 | 5 | No |  | cN0M0 |
| 52 | AST | 37 | F | Lower Extremities | Compound | 3 | - | 3 | 5 | No |  | cN0M0 |
| 53 | AST | 23 | F | Lower Extremities | Compound | 4.5 | - | 3 | 4 | No |  | cN0M0 |
| 54 | AST | 46 | F | Lower Extremities | Compound | 2.5 | - | 2 | 4 | No |  | cN0M0 |
| 55 | AST | 42 | F | Corpus | Compound | 4.5 | - | 3 | 4 | No |  | cN0M0 |
| 56 | AST | 71 | F | Upper Extremities | Compound | 2.5 | - | 2 | 3 | No |  | cN0M0 |
| 57 | AST | 71 | F | Upper Extremities | Compound | 2.5 | - | 1 | 3 | No |  | cN0M0 |
| 58 | AST | 26 | F | Lower Extremities | Compound | 9 | - | 2 | 3 | No |  | cN0M0 |
| 59 | AST | 25 | F | Corpus | Compound | 5 | - | 3 | 3 | No |  | cN0M0 |
| 60 | AST | 31 | F | Upper Extremities | Compound | 13 | - | 2 | 3 | No |  | cN0M0 |
| 61 | AST | 29 | M | Corpus | Compound | 7 | - | 1 | 6 | No |  | cN0M0 |
| 62 | AST | 17 | F | Corpus | Compound | 6 | - | 1 | 2 | No |  | cN0M0 |
| 63 | AST | 22 | F | Corpus | Compound | 4 | - | 1 | 5 | No |  | cN0M0 |
| 64 | AST | 24 | F | Upper Extremities | Compound | 6 | - | 2 | 1 | No |  | cN0M0 |
| 65 | AST | 27 | F | Upper Extremities | Compound | 7 | - | 2 | 1 | No |  | cN0M0 |
| 66 | AST | 23 | F | Corpus | Compound | 5 | - | 1 | 1 | Yes |  | cN0M0 |
| 67 | AST | 13 | M | Head/ Neck | Compound | 7 | - | 2 | 11 | No |  | cN0M0 |
| 68 | MST | 62 | M | Lower Extremities | Compound | 6 | 1.2 | 1 | 5 | No |  | pT2a  cN0M0 |
| 69 | MST | 22 | F | Corpus | Compound | 7 | 0.5 | 3 | 3 | No |  | pT1a  cN0M0 |
| 70 | MST | 55 | M | Upper Extremities | Compound | 6 | 2.5 | 3 | 3 | No |  | pT4a  pN0M0 |
| 71 | MST | 10 | F | Head/ Neck | Compound | 6 | 4.1 | 1 | 2 | No |  | pT4a cN0M0 |
| 72 | MST | 76 | M | Corpus | Compound | 6 | 0.1 | 0 | 1 | No |  | pT1a  cN0M0 |
| 73 | MST | 36 | F | Lower Extremities | Compound | 7 | 1.15 | 2 | 9 | No |  | pT2a  cN0M0 |
| 74 | MST | 86 | M | Head/ Neck | Compound | 5 | 1.12 | 1 | 1 | No |  | pT2a  cN0M0 |
| 75 | MST | 52 | M | Corpus | Compound | 5 | 0.4 | 1 | 6 | No |  | pT1a  cN0M0 |
| 76 | MST | 47 | F | Upper Extremities | Compound | 8 | 0.85 | 2 | 12 | No |  | pT2  caN0M0 |
| 77 | MST | 20 | F | Lower Extremities | Compound | 5 | 2.5 | 1 | 11 | No |  | pT3b  cN0M0 |
| 78 | MST | 42 | M | Upper Extremities | Compound | 15 | 0.8 | 3 | 8 | No |  | pT1a  cN0M0 |
| 79 | MST | 58 | M | Upper Extremities | Compound | 10 | 1.6 | 2 | 1 | No |  | pT2a  cN0M0 |
| 80 | HN | 35 | F | Corpus | Compound | 11 | - | 3 | 4 | No |  |  |
| 81 | HN | 13 | F | Head/ Neck | Compound | 7 | - | 3 | 1 | No |  |  |
| 82 | HN | NA | NA | NA | Compound | NA | - | 3 | 7 | NA |  |  |
| 83 | HN | NA | NA | NA | Compound | NA | - | 3 | 7 | NA |  |  |
| 84 | HN | NA | NA | NA | Compound | NA | - | 3 | 3 | NA |  |  |
| 85 | HN | NA | NA | NA | Compound | NA | - | 3 | 2 | NA |  |  |
| 86 | HN | NA | NA | NA | Compound | NA | - | 3 | 2 | NA |  |  |
| 87 | HN | NA | NA | NA | Compound | NA | - | 3 | 2 | NA |  |  |
| 88 | HN | NA | NA | NA | Compound | NA | - | 3 | 2 | NA |  |  |
| 89 | HN | 22 | F | Corpus | Compound | 5 | - | 3 | 3 | No |  |  |
| 90 | HN | 61 | M | Corpus | Compound | 4 | - | 3 | 1 | No |  |  |
| 91 | HN | 82 | F | Corpus | Compound | 7 | - | 3 | 1 | No |  |  |
| 92 | HN | 46 | F | Corpus | Dermal | 5 | - | 3 | 3 | No |  |  |
| 93 | HN | 36 | F | Corpus | Compound | 7 | - | 3 | 8 | No |  |  |
| 94 | HN | 20 | F | Corpus | Compound | 4 | - | 3 | 8 | No |  |  |
| 95 | HN | 12 | F | Corpus | Compound | 2 | - | 3 | 10 | No |  |  |
| 96 | HN | 36 | F | Corpus | Compound | 3 | - | 3 | 10 | No |  |  |
| 97 | HN | 10 | M | Corpus | Compound | 5 | - | 3 | 10 | No |  |  |
| 98 | HN | 15 | M | Corpus | Compound | 6 | - | 3 | 11 | No |  |  |
| 99 | HN | 28 | F | Corpus | Compound | 4 | - | 3 | 12 | No |  |  |
| 100 | HN | 22 | M | Corpus | Compound | 7 | - | 3 | 12 | No |  |  |
| 101 | HN | 17 | F | Corpus | Compound | 10 | - | 3 | 12 | No |  |  |
| 102 | HN | 28 | F | Head/ Neck | Compound | 3 | - | 3 | 13 | No |  |  |
| 103 | HN | 40 | F | Corpus | Compound | 3 | - | 3 | 13 | No |  |  |
| 104 | HN* | 28 | M | Corpus | Compound | 6 | - | 3 | 6 | No |  |  |
| 105 | BN | 22 | F | Lower Extremities | Dermal | 8 | - | 0 | 2 | No |  |  |
| 106 | BN | 48 | F | Upper Extremities | Dermal | 4 | - | 1 | 1 | No |  |  |
| 107 | BN | 66 | M | Head/ Neck | Dermal | 4 | - | 0 | 1 | No |  |  |
| 108 | BN | 69 | F | Upper Extremities | Dermal | 3 | - | 0 | 1 | No | CMM |  |
| 109 | BN | 27 | M | Upper Extremities | Dermal | 4 | - | 0 | 1 | No |  |  |
| 110 | BN | 68 | M | Corpus | Dermal | 3 | - | 0 | 1 | No |  |  |
| 111 | BN | 68 | M | Corpus | Dermal | 3 | - | 1 | 1 | No |  |  |
| 112 | BN | 28 | F | Upper Extremities | Dermal | 4 | - | 0 | 1 | No |  |  |
| 113 | BN | 41 | F | Upper Extremities | Dermal | 3 | - | 0 | 1 | No |  |  |
| 114 | BN | 19 | F | Lower Extremities | Dermal | 6 | - | 0 | 1 | No | CMM |  |
| 115 | BN | 47 | F | Upper Extremities | Dermal | 3 | - | 0 | 1 | No |  |  |
| 116 | BN | 84 | M | Lower Extremities | Dermal | 7 | - | 0 | 1 | No |  |  |
| 117 | BN | 51 | M | Lower Extremities | Dermal | 4 | - | 0 | 1 | No |  |  |
| 118 | BN | 59 | F | Upper Extremities | Dermal | 3 | - | 0 | 1 | No |  |  |
| 119 | BN | 21 | F | Head/ Neck | Dermal | 6 | - | 0 | 1 | No |  |  |
| 120 | BN | 76 | M | Head/ Neck | Dermal | 2 | - | 0 | 1 | No | CMM |  |
| 121 | BN | 78 | F | Corpus | Dermal | 4 | - | 0 | 1 | No |  |  |
| 122 | BN | 37 | M | Head/ Neck | Dermal | 15 | - | 0 | 1 | No | Squamous cell carcinoma |  |
| 123 | BN | 42 | F | Head/ Neck | Dermal | 1 | - | 0 | 2 | No |  |  |
| 124 | BN | 70 | M | Upper Extremities | Dermal | 4 | - | 0 | 2 | No |  |  |
| 125 | BN | 42 | F | Head/ Neck | Dermal | 4 | - | 1 | 2 | No |  |  |
| 126 | BN | 74 | M | Head/ Neck | Dermal | 8 | - | 1 | 2 | No |  |  |
| 127 | NCN | 51 | M | Corpus | Compound | 11 | - | 1 | 1 | No |  |  |
| 128 | NCN | 21 | F | Corpus | Junctional | 2 | - | 1 | 1 | No |  |  |
| 129 | NCN | 72 | M | Corpus | Junctional | 5 | - | 1 | 1 | No |  |  |
| 130 | NCN | 48 | F | Corpus | Compound | 10 | - | 0 | 1 | No |  |  |
| 131 | NCN | 58 | M | Corpus | Compound- | 8 | - | 2 | 1 | No |  |  |
| 132 | NCN | 58 | M | Upper Extremities | Compound | 4 | - | 2 | 1 | No |  |  |
| 133 | NCN | 71 | M | Corpus | Compound | 6 | - | 1 | 1 | No |  |  |
| 134 | NCN | 64 | F | Corpus | Compound | 6 | - | 3 | 1 | No |  |  |
| 135 | NCN | 59 | F | Corpus | Compound | 8 | - | 1 | 1 | No |  |  |
| 136 | NCN | 61 | F | Corpus | Compound | 9 | - | 1 | 1 | No |  |  |
| 137 | NCN | 43 | M | Corpus | Compound | 6 | - | 1 | 1 | No |  |  |
| 138 | NCN | 57 | M | Corpus | Compound | 10 | - | 1 | 1 | No |  |  |
| 139 | NCN | 57 | M | Corpus | Compound | 4 | - | 1 | 1 | No |  |  |
| 140 | NCN | 30 | F | Corpus | Compound | 4 | - | 0 | 1 | No |  |  |
| 141 | NCN | 54 | F | Corpus | Compound | 12 | - | 1 | 1 | No |  |  |
| 142 | NCN | 27 | M | Corpus | Compound | 6 | - | 2 | 2 | No |  |  |
| 143 | NCN | 27 | M | Corpus | Compound | 7 | - | 0 | 2 | No |  |  |
| 144 | NCN | 34 | F | Corpus | Dermal | 5 | - | 2 | 2 | No |  |  |
| 145 | NCN | 29 | M | Corpus | Compound | 6 | - | 2 | 2 | No |  |  |
| 146 | NCN | 50 | M | Head/ Neck | Dermal | 4 | - | 2 | 3 | No |  |  |
| 147 | CMM | 68 | M | Head/ Neck | Compound | 4 | 0.89 | 3 | 2 | Yes |  | pT1b  pN0M0 |
| 148 | CMM | 53 | M | Head/ Neck | Compound | 4 | 0.38 | 1 | 2 | No |  | pT1a  cN0M0 |
| 149 | CMM | 75 | M | Corpus | Compound | 16 | 1.03 | 1 | 2 | No |  | pT2a  cN0M0 |
| 150 | CMM | 71 | M | Upper Extremities | Compound | 32 | 0.48 | 3 | 2 | No |  | pT1a  cN0M0 |
| 151 | CMM | 73 | F | Head/ Neck | Compound | 13 | 0.45 | 2 | 2 | No |  | pT1a  cN0M0 |
| 152 | CMM | 88 | F | Lower Extremities | Compound | 25 | 7.2 | 0 | 2 | No |  | pT4b  cN0M0 |
| 153 | CMM | 81 | F | Corpus | Compound | 9 | 0.23 | 2 | 2 | No |  | pT1a  cN0M0 |
| 154 | CMM | 62 | F | Corpus | Compound | 7 | 0.26 | 3 | 2 | No |  | pT1a  cN0M0 |
| 155 | CMM | 76 | F | Corpus | Compound | 20 | 15.5 | 1 | 2 | Yes |  | pT4b  N3bM0 |
| 156 | CMM | 71 | M | Upper Extremities | Compound | 32 | 0.31 | 2 | 2 | No |  | pT1a  cN0M0 |
| 157 | CMM | 47 | F | Lower Extremities | Compound | 13 | 1.12 | 2 | 2 | No | BN | pT2a  pN0M0 |
| 158 | CMM | 32 | F | Head/ Neck | Compound | 8 | 0.98 | 1 | 2 | No |  | pT1b  pN0M0 |
| 159 | CMM | 80 | M | Corpus | Compound | 10 | 0.38 | 2 | 2 | No |  | pT1a  cN0M0 |
| 160 | CMM | 73 | F | Upper Extremities | Compound | 6 | 0.6 | 3 | 1 | No |  | pT1a  cN0M0 |
| 161 | CMM | 43 | F | Lower Extremities | Compound | 5 | 0.5 | 3 | 1 | No |  | pT1a  cN0M0 |
| 162 | CMM | 68 | M | Corpus | Compound | 6 | 4.5 | 1 | 1 | Yes |  | pT4a  pN2bM0 |
| 163 | CMM | 79 | M | Corpus | Compound | 25 | 1.1 | 2 | 1 | Yes |  | pT2a  pN0M0 |
| 164 | CMM | 79 | F | Lower Extremities | Compound | 8 | 0.7 | 2 | 1 | No | Lentigo maligna | pT1a  cN0M0 |
| 165 | CMM | 79 | F | Head/ Neck | Compound | 18 | 1.8 | 1 | 1 | No |  | pT2a  cN0M0 |
| 166 | CMM | 73 | M | Upper Extremities | Compound | 3 | 1.33 | 1 | 1 | No |  | pT2a  pN0M0 |

**This HN showed some lesional melanocytes with Spitzoid epithelioid and spindle shaped morphology, but characterics were insufficient for the diagnosis of a SN.*

**Legend to Table S1:**

AST = Atypical Spitz tumor, BN = Blue nevus, CMM = Conventional cutaneous malignant melanoma, f = Female, HN = Halo nevus, IP = Inflammatory Pattern, MST = Malignant Spitz tumor, m = Male, mm = Millimeter, NA = Not available, NCN = Nevocellular nevus, SN = Spitz nevus, cTNM = Clinical TNM, pTNM = Pathology TNM, y = Years. The UICC 2016 TNM classification (8^th^ edition) was used in this study.

**Table S2: Clinical and histopathological features of Spitzoid neoplasms**

|  | **Spitz Nevus**  **(SN)** | **Atypical**  **Spitz**  **Tumor**  **(AST)** | **Malignant Spitz Tumor**  **(MST),**  **Spitz Melanoma** |
| --- | --- | --- | --- |
| **Clinical features** | | | |
| **Patient age** | **Usually <20** | **Any, but usually >10** | **Any, but usually >10** |
| **Location** | **Any with limbs, face and/or neck most common** | **Any** | **Any** |
| **Architecture** | | | |
| **Diameter** | **<5mm *** | **>5mm** | **Often >10mm** |
| **Outline** | **Dome, symmetric, wedge-shaped *** | **Asymmetric** | **Asymmetric** |
| **Circumscription** | **Sharp *** | **Often poor** | **Poor** |
| **Epidermal hyperplasia** | **Present** | **Often epidermal effacement** | **Absent or epidermal effacement** |
| **Maturation with dermal depth and “zonation”** | **Present *** | **Uncommon, absent** | **Usually absent** |
| **Subcutan involvement** | **Orderly at the deep margin *** | **Frequent subcutaneous extension with “pushing” margins** | **Irregular extension at the depth with infiltrative growth** |
| **Cellular Morphology** | | | |
| **Cellular population** | **Uniform spindle and/or epithelioid *** | **Spindle and/ or epithelioid cells with increasing cytologic atypia** | **Spindle and/or epithelioid cells with increasing cytologic atypia and pleomorphism** |
| **Cytoplasm** | **Opaque/ ground glass** | **Granular** | **Granular/ mixed** |
| **Nuclei and nucleoli** | **Open, delicate chromatin pattern, uniform nucleoli** | **Heterogeneous chromatin pattern, increasingly prominent nucleoli** | **Loss of dispersed chromatin pattern, hyperchromasia, large nucleoli** |
| **Nuclear/ cytoplasmic ratio** | **Low** | **Increasingly high** | **High** |
| **Pigment** | **Superficial distribution** | **Variable** | **Variable, deep and irregularly distributed** |
| **Proliferative activity** | | | |
| **Mitotic rate** | **Absent or rare, <2/mm^2^**  **No atypical mitoses *** | **2 to 6/mm^2^**  **Deep or marginal dermal mitoses may be present** | **2 to 6/mm^2^**  **Deep or marginal dermal mitoses frequently present** |
| **Proliferative index (MIB-1/ Ki-67 IHC expression)** | **<2%** | **2-10%** | **>10%** |
| **Miscellaneous diagnostic features** | | | |
| **Ulceration** | **Absent** | **Often** | **Frequent** |
| **Kamino bodies** | **Present *** | **Absent or few** | **Typically absent** |
| **Host response** | **Inconspicuous** | **Fibroplasia; patchy mononuclear lymphocytic infiltrates in papillary dermis** | **Band-like, patchy** |
| **Other** | **Junctional clefts, teleangiectasia, transepidermal elimination of cell nests** |  |  |

Summary of histomorphological characteristics in the diagnosis of Spitzoid neoplasms with * highlighting most helpful diagnostic features in the differential of Spitz nevus (SN), atypical Spitz tumor (AST) and Spitz melanoma, i.e. malignant Spitz tumor (MST). Adapted from [7, 31] and reviewed in [33].

**Table S3: Overview of antibodies used for immunohistochemistry (IHC) staining**

| **Antibody** | **Company** | **Clone** | **Dilution** | **Staining characteristics** | **Positive control** | **Detected cell** |
| --- | --- | --- | --- | --- | --- | --- |
| **Anti-CD3** | DAKO | IR503 | RTU | M | Tonsil, LN | T-cells |
| **Anti-CD4** | DAKO | IR649 (4B12) | RTU | M | Tonsil, LN | T-helper cells and histiocytic cells |
| **Anti-CD8** | DAKO | IR623 (C8/144B) | RTU | M | Tonsil, LN | Cytotoxic T-cells |
| **Anti-CD68** | DAKO | IR609 (KP1) | RTU | C, M | Tonsil, LN | Histiocytes |
| **Anti-CD138** | DAKO | IR642 (MI15) | RTU | M | Tonsil, LN, epithelium and adnexal structures | Plasma cells |
| **Anti-Granzyme B** | DAKO | GrB-7 | 1 in 50 | C | Tonsil, LN | Cytotoxic T-cells and NK- cells |
| **Anti-TIA-1** | Beckman coulter | 2G9A10F5 | RTU | C | Tonsil, LN | Cytotoxic T-cells and NK- cells |

**Legend to Table S3:**

C = Cytoplasm, LN = Lymph node, M = Membrane, RTU = Ready to use, TIA-1 = T cell intracellular antigen-1

**Table S4:** **Statistical analysis of level of significance for the different diagnostic groups**

| **Melanocytic diagnostic category** | **SN** | **AST** | **MST** |  | **HN** | **BN** | **NCN** | **CMM** |
| --- | --- | --- | --- | --- | --- | --- | --- | --- |
| **Age (y)** | | | | | | | | |
| **SN** |  | 0.197 | **0.003**  0.053 |  | 0.444 | **<0.001**  **<0.001** | **<0.001**  **<0.001** | **<0.001**  **<0.001** |
| **AST** | 0.197 |  | 0.097 |  | 0.586 | **0.007**  0.150 | **0.003**  0.068 | **<0.001**  **<0.001** |
| **MST** | **0.003**  0.053 | 0.097 |  |  | **0.038**  0.790 | 0.576 | 0.755 | **0.009**  0.180 |
| **HN** | 0.444 | 0.586 | **0.038**  0.790 |  |  | **0.002**  **0.040** | **0.003**  0.060 | **<0.001**  **<0.001** |
| **BN** | **<0.001**  **<0.001** | **0.007**  0.150 | 0.576 |  | **0.002**  **0.040** |  | 0.614 | **0.004**  0.089 |
| **NCN** | **<0.001**  **<0.001** | **0.003**  0.068 | 0.755 |  | **0.003**  0.06 | 0.614 |  | **0.0002**  **0.0037** |
| **CMM** | **<0.001**  **<0.001** | **<0.001**  **<0.001** | **0.009**  0.18 |  | **<0.001**  **<0.001** | **0.004**  0.089 | **0.0002**  **0.0037** |  |
| **Gender** | | | | | | | | |
| **SN** |  | 0.126 | 0.108 |  | 1.000 | 0.298 | 0.057 | 0.409 |
| **AST** | 0.126 |  | **0.014**  0.295 |  | 0.402 | **0.037**  0.767 | **0.006**  0.116 | **0.036**  0.766 |
| **MST** | 0.108 | **0.014**  0.295 |  |  | 0.136 | 0.721 | 1.000 | 0.716 |
| **HN** | 1.000 | 0.402 | 0.136 |  |  | 0.332 | 0.059 | 0.328 |
| **BN** | 0.298 | **0.037**  0.767 | 0.721 |  | 0.332 |  | 0.374 | 1.000 |
| **NCN** | 0.057 | **0.006**  0.116 | 1.000 |  | 0.059 | 0.374 |  | 0.527 |
| **CMM** | 0.409 | **0.036**  0.766 | 0.716 |  | 0.328 | 1.000 | 0.527 |  |
| **Anatomical Site** | | | | | | | | |
| **SN** |  | 0.223 | 0.796 |  | **<0.001**  **<0.001** | 0.321 | **<0.001**  **<0.001** | 0.427 |
| **AST** | 0.223 |  | 0.730 |  | **<0.001**  **0.015** | 0.103 | **0.003**  0.06 | 0.512 |
| **MST** | 0.796 | 0.730 |  |  | **<0.001**  **0.005** | 0.705 | **<0.001**  **0.008** | 0.800 |
| **HN** | **<0.001**  **<0.001** | **<0.001**  **0.015** | **<0.001**  **0.005** |  |  | **<0.001**  **<0.001** | 0.792 | **0.0027**  0.056 |
| **BN** | 0.321 | 0.103 | 0.705 |  | **<0.001**  **<0.001** |  | **<0.001**  **<0.001** | 0.392 |
| **NCN** | **<0.001**  **<0.001** | **0.003**  0.060 | **<0.001**  **0.008** |  | 0.792 | **<0.001**  **<0.001** |  | **0.0013**  **0.027** |
| **CMM** | 0.427 | 0.512 | 0.800 |  | **0.0027**  0.056 | 0.392 | **0.0013**  **0.027** |  |
| **Histological features** | | | | | | | | |
| **SN** |  | 0.098 | 0.323 |  | 0.067 | **<0.001**  **<0.001** | 1.000 | 0.057 |
| **AST** | 0.098 |  | - |  | 1.000 | **<0.001**  **<0.001** | 0.240 | - |
| **MST** | 0.323 | - |  |  | 1.000 | **<0.001**  **<0.001** | 0.387 | - |
| **HN** | 0.067 | 1.000 | 1.000 |  |  | **<0.001**  **<0.001** | 0.219 | - |
| **BN** | **<0.001**  **<0.001** | **<0.001**  **<0.001** | **<0.001**  **<0.001** |  | **<0.001**  **<0.001** |  | **<0.001**  **<0.001** | **<0.001**  **<0.001** |
| **NCN** | 1.000 | 0.240 | 0.387 |  | 0.219 | **<0.001**  **<0.001** |  | **<0.001**  **<0.001** |
| **CMM** | 0.057 | - | - |  | - | **<0.001**  **<0.001** | **<0.001**  **<0.001** |  |
| **Lesion diameter (mm)** | | | | | | | | |
| **SN** |  | 0.063 | **0.0002**  **0.004** |  | 0.055 | 0.877 | **0.0003**  **0.005** | **<0.001**  **<0.001** |
| **AST** | 0.063 |  | 0.107 |  | 0.973 | 0.152 | 0.263 | **0.003**  0.06 |
| **MST** | **0.0002**  **0.004** | 0.107 |  |  | 0.086 | **0.002**  **0.048** | 0.635 | 0.078 |
| **HN** | 0.055 | 0.973 | 0.086 |  |  | 0.146 | 0.184 | **0.002**  **0.045** |
| **BN** | 0.877 | 0.152 | **0.002**  **0.048** |  | 0.146 |  | **0.005**  0.1 | **<0.001**  **0.0016** |
| **NCN** | **0.0003**  **0.005** | 0.263 | 0.635 |  | 0.184 | **0.005**  0.100 |  | **0.021**  0.440 |
| **CMM** | **<0.001**  **<0.001** | **0.003**  0.060 | 0.078 |  | **0.002**  **0.045** | **<0.001**  **0.0016** | **0.021**  0.440 |  |
| **IP score** | | | | | | | | |
| **SN** |  | **0.020**  0.411 | **0.019**  0.391 |  | **<0.001**  **<0.001** | **<0.001**  **<0.001** | **0.030**  0.639 | **0.018**  0.374 |
| **AST** | **0.020**  0.411 |  | 0.595 |  | **<0.001**  **<0.001** | **<0.001**  **<0.001** | 0.067 | 0.956 |
| **MST** | **0.019**  0.391 | 0.595 |  |  | **<0.001**  **<0.001** | **<0.001**  **<0.001** | 0.538 | 0.948 |
| **HN** | **<0.001**  **<0.001** | **<0.001**  **<0.001** | **<0.001**  **<0.001** |  |  | **<0.001**  **<0.001** | **<0.001**  **<0.001** | **<0.001**  **<0.001** |
| **BN** | **<0.001**  **<0.001** | **<0.001**  **<0.001** | **<0.001**  **<0.001** |  | **<0.001**  **<0.001** |  | **<0.001**  **<0.001** | **<0.001**  **<0.001** |
| **NCN** | **0.030**  0.639 | 0.067 | 0.538 |  | **<0.001**  **<0.001** | **<0.001**  **<0.001** |  | 0.278 |
| **CMM** | **0.018**  0.374 | 0.956 | 0.948 |  | **<0.001**  **<0.001** | **<0.001**  **<0.001** | 0.278 |  |
| **Mean follow up (y)** | | | | | | | | |
| **SN** |  | 0.197 | 0.935 |  | 0.201 | **<0.001**  **<0.001** | **<0.001**  **<0.001** | **<0.001**  **<0.001** |
| **AST** | 0.197 |  | 0.545 |  | 0.094 | **<0.001**  **<0.001** | **<0.001**  **<0.001** | **0.0003**  **0.006** |
| **MST** | 0.935 | 0.545 |  |  | 0.344 | **0.0003**  **0.007** | **0.0007**  **0.015** | **0.008**  0.171 |
| **HN** | 0.201 | 0.094 | 0.344 |  |  | **<0.001**  **<0.001** | **<0.001**  **<0.001** | **<0.001**  **<0.001** |
| **BN** | **<0.001**  **<0.001** | **<0.001**  **<0.001** | **0.0003**  **0.007** |  | **<0.001**  **<0.001** |  | 0.799 | **0.006**  0.132 |
| **NCN** | **<0.001**  **<0.001** | **<0.001**  **<0.001** | **0.0007**  **0.015** |  | **<0.001**  **<0.001** | 0.799 |  | **0.022**  0.469 |
| **CMM** | **<0.001**  **<0.001** | **0.0003**  **0.006** | **0.008**  0.171 |  | **<0.001**  **<0.001** | **0.006**  0.132 | **0.022**  0.469 |  |

**Table S4:** The first value in a cell shows the p-value per analyzed condition without post-hoc correction. In case of significance (p < 0.05; bold) the Bonferroni analysis was applied as post-hoc correction and p-values are given as second values in the according cell. For the parameters age, diameter and follow up the Kruskal-Wallis analysis was used. For the categorical variables (inflammatory pattern (IP) score, gender, anatomical site, histological features) Fisher’s exact test was used. A ’-‘ in a cell indicates that analyzed groups have identical distribution of the investigated feature.

**Legend to Table S4:** AST = Atypical Spitz tumor, BN = Blue nevus, HN = Halo nevus, CMM = Cutaneous malignant melanoma, IP = Inflammatory pattern, mm = Millimeter, MST = Malignant Spitz tumor, N = Number, NA = Not available, NCN = Nevocellular nevus, SEM = Standard error of the mean, SD = Standard deviation, y = Years
